# Genomic comparisons reveal biogeographic and anthropogenic impacts in the koala (*Phascolarctos cinereus*): a dietary-specialist species distributed across heterogeneous environments

**DOI:** 10.1038/s41437-018-0144-4

**Published:** 2018-09-12

**Authors:** Shannon R. Kjeldsen, Herman W. Raadsma, Kellie A. Leigh, Jennifer R. Tobey, David Phalen, Andrew Krockenberger, William A. Ellis, Emily Hynes, Damien P. Higgins, Kyall R. Zenger

**Affiliations:** 1 Centre for Sustainable Tropical Fisheries and Aquaculture, James Cook University, Townsville, QLD 4811 Australia; 2 Sydney School of Veterinary Science, Faculty of Science, The University of Sydney, Camden, Private Mail Bag 4003, Narellan, NSW 2570 Australia; 3 Science for Wildlife, PO Box 286, Cammeray, NSW 2062 Australia; 4 San Diego Zoo Institute for Conservation Research, Escondido, CA 92027 USA; 5 Centre for Tropical Biodiversity and Climate Change, Division of Research and Innovation, James Cook University, Cairns, QLD 4878 Australia; 6 School of Agriculture and Food Science, The University of Queensland, St Lucia, QLD 4072 Australia; 7 Ecoplan Australia, PO Box 968, Torquay, VIC 3228 Australia; 8 Sydney School of Veterinary Science, Faculty of Science, The University of Sydney, Sydney, NSW 2006 Australia

**Keywords:** Genetic variation, Conservation biology, Ecological genetics, Population genetics, Phylogenetics

## Abstract

The Australian koala is an iconic marsupial with highly specific dietary requirements distributed across heterogeneous environments, over a large geographic range. The distribution and genetic structure of koala populations has been heavily influenced by human actions, specifically habitat modification, hunting and translocation of koalas. There is currently limited information on population diversity and gene flow at a species-wide scale, or with consideration to the potential impacts of local adaptation. Using species-wide sampling across heterogeneous environments, and high-density genome-wide markers (SNPs and PAVs), we show that most koala populations display levels of diversity comparable to other outbred species, except for those populations impacted by population reductions. Genetic clustering analysis and phylogenetic reconstruction reveals a lack of support for current taxonomic classification of three koala subspecies, with only a single evolutionary significant unit supported. Furthermore, ~70% of genetic variance is accounted for at the individual level. The Sydney Basin region is highlighted as a unique reservoir of genetic diversity, having higher diversity levels (i.e., Blue Mountains region; AvHe^corr^=0.20, PL% = 68.6). Broad-scale population differentiation is primarily driven by an isolation by distance genetic structure model (49% of genetic variance), with clinal local adaptation corresponding to habitat bioregions. Signatures of selection were detected between bioregions, with no single region returning evidence of strong selection. The results of this study show that although the koala is widely considered to be a dietary-specialist species, this apparent specialisation has not limited the koala’s ability to maintain gene flow and adapt across divergent environments as long as the required food source is available.

## Introduction

Specialist species evolve in stable environments to exploit available niches. However, specific adaptation to these niches, whether they be dietary or habitat specialisation, can make them more vulnerable to stochastic change than generalist species. Local persistence and dispersal rates of specialist species are strongly influenced by degree and type of ecological specialisation (Li et al. 2014; Kierepka et al. 2016), and capacity to adapt to habitat change (Dennis et al. 2011; Hardy and Otto 2014). The level of ecological specialisation can predict how well a species might survive in a recently modified landscape, and also how the species may adapt over time, which plays an important role in understanding species diversification (Dennis et al. 2011; Hardy and Otto 2014).

Specialist species often occupy smaller, more fragmented habitats and have smaller effective population sizes than their generalist counterparts (Horsák et al. 2012).Therefore, species with narrow ecological requirements are expected to be highly sensitive to further habitat loss and fragmentation (Franzén et al. 2012; Kierepka et al. 2016). This leads to reduced gene flow and highly structured populations, which can increase the effects of random genetic drift, genetic bottlenecks, inbreeding and/or extinction events (Dennis et al. 2011; Li et al. 2014). Loss of genetic diversity and connectivity via these processes limits the evolutionary potential and can alter the evolutionary trajectory of the species.

Patterns of genetic differentiation vary considerably across specialist and generalist species (Packer et al. 2005). Specialisation in one dimension may lead to generalisation in another, or it may be context dependent, and specialisation may be restricted temporally or developmentally (reviewed by Li et al. 2014). Differences in selection pressures between populations due to ecological heterogeneity are potent drivers of evolutionary change. Understanding the genetic impacts of species-specific sensitivities to habitat changes is a crucial step towards formulating reliable predictions of species persistence and population structuring, which are valuable for understanding evolutionary processes, and informing conservation and management strategies (Murphy et al. 2011; Khimoun et al. 2016).

**Fig. 1:**
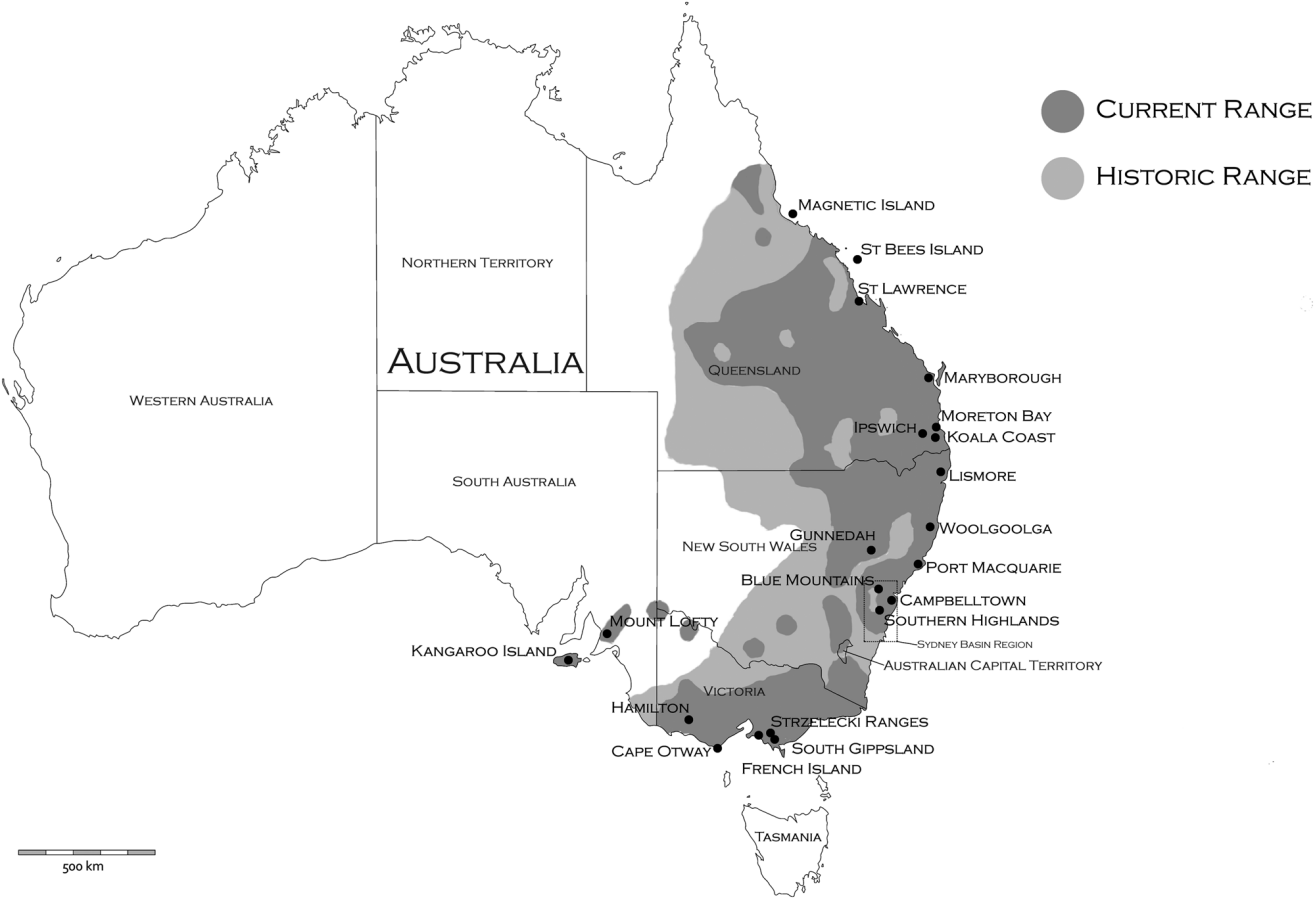
Distribution and current sampling range of *Phascolarctos cinereus* (currently and historically). Adapted from distribution map created by Strahan et al. (1995)

The koala is a marsupial with a specialised folivorous diet that can be found across much of the eastern coast of Australia (Fig. 1). Koalas utilise up to 120 different species of tree across their distribution, but primary food tree species can be as few as two within a particular area (Melzer et al. 2000; Tucker et al. 2007). Furthermore, variability in chemical profiles even within a single eucalypt species can affect koala browsing preferences in different regions (Moore et al. 2005). This specialised diet limits their potential habitat to regions able to support these eucalypt species. Despite the koala’s specialist dietary and habitat requirements, they are distributed across a vast range of environments and climatic zones from subalpine forests in Victoria (VIC) to subtropical forests in far north Queensland (QLD) (Melzer et al. 2000; Penn et al. 2000; Phillips 2000). However, the contemporary distribution of the koala is not continuous across this range due to habitat fragmentation. As a consequence of translocations, koalas now occur outside their natural range. These areas include many Victorian and QLD Islands, and South Australia (SA) (Melzer et al. 2000).

As is expected of an animal spanning a large range of varied habitats, the koala exhibits morphological differences (e.g., body size, pelage and skull characteristics) between its northernmost and southernmost populations, with intermediate phenotypes in the middle of its range (Black et al. 2014; Briscoe et al. 2015). The phenotypic variation across the species range, along with differences in skull morphology, historically led to koalas being classified into three separate subspecies (*P. c. adustus, P. c. cinereus* and *P. c. victor*). This classification was first described in the early 20th century, based on skull morphology and skins alone (Thomas 1923; Troughton 1935, 1941). There currently is no supporting genetic evidence for this taxonomic delineation. Genetic studies have attempted to understand taxonomic relationships using mitochondrial DNA (Houlden et al. 1996, 1999; Neaves et al. 2016; Tsangaras et al. 2012), with results indicating a lack of support for the current subspecies classification. This outcome was also observed in a preliminary genome-wide single-nucleotide polymorphism (SNP) study, which again suggested that the current taxonomic classification should be re-addressed (Kjeldsen et al. 2016).

Determining appropriate species-wide management actions for the koala has been challenging due to limited information on broad-scale population connectivity and genetic structure across divergent landscapes. The current patterns of genetic diversity of koalas are likely to have been influenced by human activities, including clearing of habitat, hunting and translocations. The conservation status of koalas varies across its distribution. Under Australian Federal law, the koala is classified as vulnerable in QLD, New South Wales (NSW) and Australian Capital Territory (ACT) but is not listed in VIC and SA. This dichotomy in conservation status is a reflection of the different overall population histories of koalas in these states and territories. Koala population declines have been observed across NSW and south east QLD, whereas some populations in VIC and SA are considered to be over-abundant. To prevent defoliation of preferred food trees, and subsequent starvation of koalas in these over-abundant populations, they are actively managed through translocation and fertility control (Menkhorst 2008; McAlpine et al. 2015; Whisson et al. 2016).

Anthropogenic influences have been particularly strong in the southern states of VIC and SA where the koala has a unique management history. A fur trade was established in the late 1800s and this, in combination with habitat destruction and wildfire, led to a dramatic decline in koala numbers (Menkhorst 2008). By the 1920s, only a few remnant southern populations remained (Menkhorst 2008). However, concurrent with population declines on the mainland, koalas were introduced to islands outside their normal range, most notably French Island, which was founded by as few as two or three individuals sourced from mainland VIC (Menkhorst 2008; Houlden et al. 1996; Lewis 1934, 1954; Warneke 1978). The growth rate of the French Island population was so rapid that severe defoliation was observed within a short period of time (Martin and Handasyde 1999; Menkhorst 2008). From 1923 until the 21st century, koalas have been translocated from French Island to alleviate browse pressure. These translocations have created new populations, including Kangaroo Island, which was reported as being established by 18 adult French Island animals (Masters et al. 2004). In almost a century of active management, koalas have been reintroduced to over 250 locations across VIC (Menkhorst 2008). These management actions may have secured the future of koalas in VIC, but at the cost of genetic diversity (Martin and Handasyde 1999; Menkhorst 2008). Although population reductions may not have been as drastic in northern regions, within QLD, several islands now support remnant or introduced koala populations (see Lee et al. 2012). During the 1930s, the St Bees Island koala population in central QLD was founded from as few as 12–17 individuals (Berck 1995), whereas the north QLD Magnetic Island population was established from at least 18 individuals (Hrdina and Gordon 2004).

It has been demonstrated that substantial population size reductions, and genetic swamping due to translocations, of remnant populations has influenced the genetic diversity of koalas in Australia (Menkhorst 2008; Lee et al. 2012b; Wedrowicz et al. 2018). Despite these influences, a preliminary study by Kjeldsen et al. (2016) revealed that population diversity is still highly variable across the species geographic range, indicating that diversity may not be reduced in all regions. A number of other studies have also attempted to understand how the koala’s ecological history has influenced genetic diversity (Menkhorst 2008; Houlden et al. 1996, 1999). However, many of these were conducted on a local level, which limited their interpretation across the species distribution, or the investigators did not have access to genome-wide genetic markers, which is important for examining adaptive variation (Fowler et al. 2000; Houlden et al. 1996, 1999; Lee et al. 2010, 2012; Neaves et al. 2016; Timms et al. 1993; Wilmer et al. 1993).

To date, there is limited information on connectivity and gene flow at a species-wide scale, while considering the potential impacts of local adaptation (Kjeldsen et al. 2016; Menkhorst 2008; Houlden et al. 1996, 1999). Given the specialist nature of the koala distributed across divergent landscapes, it is important to understand how ecological and anthropogenic influences impact koala populations. Identifying levels of gene flow, genetic diversity and signatures of local adaptation will help inform at what geographical and/or ecological scale management should be implemented. In this study, using the most comprehensive genomic dataset to date, we aim to: (1) examine the levels of genetic diversity in wild koala populations across the species range, (2) assess patterns of contemporary genetic structuring and connectivity between populations and bioregions and (3) provide insights into historical divergence among populations through phylogenetic reconstructions using genome-wide markers.

## Methods

### Sampling and DNA extraction

To ensure both natural and introduced koala populations were sampled across different bioregions and throughout the species distribution, a total of 21 representative regions (Fig. 1) were sampled opportunistically from wild koala populations across eastern-Australia (equating to 800 tissue or blood samples, see acknowledgements for further details). Tissue samples were preserved in 70% ethanol, whereas whole-blood samples were allowed to clot, before being stored at −20 °C. All DNA samples were extracted using a modified CTAB/Cholorform-Isoamyl method (Adamkewicz and Harasewych 1996) and further purified using a Sephadex G-50 approach (GE Healthcare Life Sciences 2000) to ensure no inhibitors were carried through to downstream genotyping.

### Library preparation and sequencing

All samples were sequenced and genotyped using DArTseq™ genotyping technology at Diversity Arrays Technology, Canberra, Australia (Jaccoud et al. 2001, Kilian et al. 2012). Briefly, approximately 100 ng (2 µL) of each sample was digested with a combination of both a frequent and rare cutting restriction enzyme, *Pts*I and *Sph*I, and unique barcode sequences simultaneously ligated onto the ends of each resulting fragment (Kilian et al. 2012). The *Pst*I-specific adaptor incorporated an Illumina flow-cell attachment region, coupled with a primer sequence, and unique barcode, with the reverse *Sph*I-specific adaptor containing a second Illumina flow-cell attachment sequence to facilitate bridge amplification (Lind et al. 2017; Schultz et al. 2018). A minimum of 15% random technical replicates were included in all genotyping batches for downstream quality control. Each sample was individually amplified using proprietary barcode and adaptor sequences, with only fragments containing both *Pst*I and *Sph*I cut sites being amplified for sequencing, before being purified using a Qiagen PCR clean-up kit (Werle et al. 1994). Each sample was checked visually on an agarose gel to ensure complete digestion and conformity to a uniform range of fragment sizes. Samples, which displayed incomplete digestion or a downshifted digestion pattern, were removed from the library and not carried forward. Using approximately 10 µL of each sample, batches of 288 samples were pooled for sequencing on a single flow-cell lane on the Illumina HiSeq2500 for 77 cycles.

### Quality control and initial SNP calling

DArTseq™ genotyping technology generates two independent marker types – SNPs and presence–absence variants (PAV, dominant loci) identified from restriction site-associated (RAD) fragments. SNPs were used for both population and phylogenetic analyses, whereas PAVs were only used in phylogenetic reconstructions. To ensure the highest quality loci were carried through to downstream analysis, the following sequence quality control and filtering measures were conducted. Raw sequence data in a fastq file format were obtained, and de-multiplexed according to individual barcodes. Each read was assessed for overall quality, and any reads containing base pair Q-scores <30 were removed. All reads were checked against existing sequences in the DArTdb database (Sivasankaran et al. 1993) and also against viral and bacterial databases to assess contamination. If any contamination was identified, those reads were removed from the dataset.

SNP and PAV calling were performed using the *DArTsoft14* algorithm within the KDCompute pipeline developed by Diversity Arrays Technology (http://www.kddart.org/kdcompute.html). KDCompute SNP calling was carried out by creating clusters of identical reads at a population (or dataset) level, with three nucleotide mismatches allowed, then similar clusters were matched together to identify polymorphisms within sequence reads as described by Wenzl et al. (2004) and Lind et al. (2017). All monomorphic and tri-allelic loci were removed from the SNP dataset. In order for a SNP to be called by KDCompute, both homozygous and heterozygous forms were required to be present within the entire dataset. Following SNP identification, the following metrics were provided with the dataset; homozygote and heterozygote numbers, call rate, allele frequency, polymorphic information content (PIC), average PIC across all individuals, average SNP count, average read depth and reproducibility (based on random replicates). Further filtering was conducted using custom python scripts (Steinig 2016, https://github.com/esteinig/dartQC). SNPs with an average read depth of <10 (Nielsen et al. 2011) and MAF (minor allele frequency) of <0.01 were removed from the dataset. If multiple SNPs were observed within a sequence read, only the SNP with the highest call rate across individuals and MAF were retained. Any sample/locus with a reproducibility of <95% and a call rate of <70% were removed from the dataset. To capture independent loci, SNPs in linkage disequilibrium (LD; *r*^2^ > 0.2) were identified across all populations using PLINK (Purcell et al. 2007). From pairs of loci in LD, the SNP with the lower call rate and MAF value was removed. Each SNP was also assessed for deviation from Hardy–Weinberg equilibrium (HWE) at a population level using Arlequin v3.5 (Excoffier and Lischer 2010), and if a SNP significantly deviated from HWE (*P* < 0.0001) in all populations, it was removed from the working dataset, SNPs, which deviated in only a single population, but were in HWE across the entire dataset were retained. Loci putatively identified as sex linked were removed from the final SNP dataset according to Kjeldsen et al. (2016). PAV loci are scored as 0 or 1 and were extracted in silico from sequences obtained from genomic representations (Lind et al. 2017). PAV loci are based on a range of DNA variations in the restriction enzyme recognition sites. PAV loci were filtered manually and only PAV markers with a call rate of 100%, MAF > 0.02 across the dataset and technical reproducibility of 100% were retained, according to Lal et al. (2016).

To further assess the distribution of SNP and PAV markers across the koala genome, all sequence reads were mapped back to the koala genome (Assembly Accession number: GCA_002099425.1, Genbank accession number: MSTS00000000.1), using the fast read mapper function in Bowtie 2 (Langmead and Salzberg 2012). Only a single read was retained if multiple reads mapped back to the same genomic region, markers were selected based on being evenly spread across each genomic scaffold, where only the marker with the highest call rate was retained.

### Identification of signatures of selection

In order to identify loci that are under selection, outlier analyses were conducted using a Bayesian approach, implemented within the program BayeScan 2.01 (Foll 2012). All South Australian and Victorian populations were excluded from these analyses, as population bottlenecks are known to affect outlier detection (Thornton and Jensen 2007). Analyses were conducted using 1:10 prior odds for a neutral model and all other parameters left as default (20 pilot runs of 5000 iterations followed by 100,000 iterations with an additional burn-in of 50,000). Outliers were identified with a false discovery rate (FDR) of 0.001 and 0.01 using the Bayescan 2.01 function, plot_R.r. Both directional (alpha > 0) and balancing or purifying (alpha ≤ 0) loci under selection were putatively identified. To help understand the impact of directional selection upon genetic relationships, the 1-proportion of shared allele distance matrix was calculated using the “propShared” function in *adegenet* (Jombart 2008) using both neutral and outlier loci. Individual relationships were then visualised using neighbour-joining (NJ) trees constructed in MEGA6 (Tamura et al. 2013; Lal et al. 2016). Population pairwise *F*_ST_ values were also calculated independently for both neutral and directional outlier loci using Weir and Cockerham’s (1984) unbiased approach based on 999 permutations within the Genalex v6.502 analysis package (Peakall and Smouse 2006). To investigate genetic signatures of selection among heterogeneous environments, populations were assigned to their specific bioregion according to Interim Biogeographic Regionalisation for Australia map (IBRA version 7, 2012; Table 1). Following the subtraction of neutral *F*_ST_ values from outlier *F*_ST_ values (to estimate the level of selective forces alone), the average within- and between-bioregion *F*_ST_ values were calculated.

**Table 1:** Diversity indices including: populations name and region, number of samples (*n*), bioregion (based on IBRA version 7, 2012), corrected expected heterozygosity (He^corr^), observed heterozygosity (Ho), percentage of polymorphic loci (%PL), inbreeding coefficient (*F*_IS_), average *F*_ST_ between a single population and all others (AvF_ST_), number of private alleles per population (#Ap), frequency of rare alleles (MAF < 0.05) per population (Ar), standardised multilocus heterozygosity (sMLH) and internal relatedness (IR)

	Population	*n*	Bioregion	He (corr) ± SE	Ho ± SE	% PL	Fis ± SE	avFst ± SD	# Ap	Ar ± SE	sMLH ± SD	IR ± SD
1	Magnetic Island (MI)	20	BBN	0.14 ± 0.00	0.14 ± 0.00	47.8%	0.01 ± 0.00	0.3 ± 0.12	0	0.07 ± 0.00	1.02 ± 0.05	0.57 ± 0.03
2	St Bees Island (SB)	21	CMC	0.14 ± 0.00	0.14 ± 0.00	53.8%	−0.03 ± 0.00	0.32 ± 0.13	0	0.08 ± 0.00	1.07 ± 0.36	0.55 ± 0.14
3	St Lawrence (SL)	18	CMC	0.18 ± 0.00	0.16 ± 0.00	60.7%	0.07 ± 0.00	0.21 ± 0.11	0	0.11 ± 0.00	1.1 ± 0.16	0.55 ± 0.10
4	Maryborough (M)	14	SEQ	0.15 ± 0.00	0.14 ± 0.00	45.2%	0 ± 0.00	0. 29 ± 0.12	0	0.06 ± 0.00	1.02 ± 0.09	0.57 ± 0.04
5	Moreton Bay (MB)	8	SEQ	*	*	*	*	*	*	*	*	*
6	Koala Coast (KC)	20	SEQ	0.17 ± 0.00	0.16 ± 0.00	59.6%	0.03 ± 0.00	0.23 ± 0.11	0	0.1 ± 0.00	1.11 ± 0.09	0.55 ± 0.06
7	Ipswich (I)	22	SEQ	0.19 ± 0.00	0.17 ± 0.00	68.9%	0.07 ± 0.00	0.21 ± 0.12	0	0.13 ± 0.00	1.2 ± 0.13	0.50 ± 0.07
8	Lismore (LI)	77	SEQ	0.17 ± 0.00	0.15 ± 0.00	74.5%	0.11 ± 0.00	0.24 ± 0.11	5	0.15 ± 0.00	1.07 ± 0.11	0.55 ± 0.05
9	Woolgoolga (W)	9	NNC	*	*	*	*	*	*	*	*	*
10	Gunnedah (GD)	57	BBS	0.16 ± 0.00	0.15 ± 0.00	64.6%	0.06 ± 0.00	0.26 ± 0.08	7	0.11 ± 0.00	1.03 ± 0.15	0.49 ± 0.10
11	Port Macquarie (PM)	85	NNC	0.18 ± 0.00	0.17 ± 0.00	80.9%	0.06 ± 0.00	0.23 ± 0.1	5	0.18 ± 0.01	1.22 ± 0.19	0.58 ± 0.19
12	Blue Mountains (BM)	19	SYB	0.20 ± 0.00	0.18 ± 0.00	68.6%	0.1 ± 0.00	0.15 ± 0.06	0	0.18 ± 0.01	0.99 ± 0.35	0.63 ± 0.11
13	Campbelltown (CT)	119	SYB	0.15 ± 0.00	0.14 ± 0.00	82.5%	0.03 ± 0.00	0.32 ± 0.08	2	0.12 ± 0.00	1.1 ± 0.27	0.53 ± 0.11
14	Southern Highlands (SH)	25	SYB	0.18 ± 0.00	0.15 ± 0.00	64.0%	0.08 ± 0.00	0.22 ± 0.07	0	0.09 ± 0.00	1.06 ± 0.15	0.56 ± 0.05
15	South Gippsland (SG)	17	SCP	0.11 ± 0.00	0.1 ± 0.00	37.7%	−0.01 ± 0.00	0.29 ± 0.12	0	0.04 ± 0.00	0.73 ± 0.06	0.70 ± 0.09
16	Strzelecki (SZ)	19	SCP	0.11 ± 0.00	0.11 ± 0.00	39.4%	−0.01 ± 0.00	0.27 ± 0.1	0	0.04 ± 0.00	0.76 ± 0.16	0.68 ± 0.09
17	French Island (FI)	39	SCP	0.10 ± 0.00	0.11 ± 0.00	49.1%	0.09 ± 0.00	0.36 ± 0.08	0	0.09 ± 0.00	0.61 ± 0.24	0.80 ± 0.15
18	Cape Otway (CO)	28	SCP	0.12 ± 0.00	0.11 ± 0.00	53.7%	0.08 ± 0.00	0.23 ± 0.1	0	0.07 ± 0.00	0.67 ± 0.34	0.75 ± 0.04
19	Hamilton (H)	4	VIM	**	**	**	**	**	**	**	**	**
20	Mt Lofty (ML)	23	EYB	0.13 ± 0.00	0.12 ± 0.00	60.2%	0.01 ± 0.01	0.42 ± 0.09	0	0.04 ± 0.00	0.59 ± 0.12	0.76 ± 0.12
21	Kangaroo Island (KI)	14	KAN	0.13 ± 0.00	0.09 ± 0.00	44.6%	0.19 ± 0.01	0.3 ± 0.12	0	0.04 ± 0.00	0.59 ± 0.33	0.83 ± 0.11

Metrics for populations with *n* < 10 should be considered with caution due to potential subsampling effects

*Metrics omitted due to low sample size

Finally, to assess if any putatively identified outlier loci were associated with genic regions, all identified outlier SNP sequence reads was mapped to the koala genome (Assembly Accession number: GCA_002099425.1, Genbank accession number: MSTS00000000.1). Additionally, the region surrounding each marker (±2000 bp) was extracted and subsequently compared with publicly available genomic databases to identify any markers, which may be closely linked to functional regions (Supplementary Table 4).

### Population-specific genetic diversity

To evaluate the level of genetic diversity within and between populations/regions, standard diversity indices (based on putatively neutral SNP loci) including average expected heterozygosity corrected for sample size (He^corr^), average observed heterozygosity (Ho), inbreeding coefficient (*F*_IS_), number of private alleles (Ap) and rare alleles (Ar; MAF < 5%) were calculated through the Genalex v6.502 analysis package (Peakall and Smouse 2006). To assess individual genome-wide diversity and inbreeding measures, standardised multilocus heterozygosity (sMLH), and internal relatedness (IR) were calculated for all individuals using the R package *Rhh* (Alho et al. 2010). Koalas are largely solitary animals, with structured social hierarchies, and display highly variable home range sizes (0.4–300 ha) (Davies et al. 2013). In order to identify closely related individuals, relatedness was assessed using a maximum likelihood (ML) approach in MLrelate (Kalinowski et al. 2006). Individuals returning high relatedness values (>0.25) were identified within each population to assist in the interpretation and account for potential bias in the data (Hansen et al. 1997).

### Population structure

Based on putative neutral SNP loci, pairwise genetic divergence between populations was evaluated using Weir and Cockerham’s unbiased *F*-statistics (*F*_ST_) (Weir and Cockerham 1984) and Nei’s unbiased genetic distance (Nei 1978) in Genalex v6.502 (Peakall and Smouse 2006). *F*_ST_ values were also calculated using Wright’s *F*_ST_ approach in Arlequin v3.5 (Excoffier et al. 2005; Wright 1965), and Meirman’s approach in GenoDive (Meirmans and Van Tienderen 2004). However, as the Meirman’s values were similar, and the Meirman’s approach returned some negative, nonsignificant values, only Weir and Cockerham’s unbiased *F*_ST_ values are presented here (Table 2, Supplementary Table 2). Genotypic relationships between individuals were visualised using the NetView R program (Neuditschko et al. 2012; Steinig et al. 2015) at multiple k-NN values (k-NN = 10–100). Optimisation of k-NN values was performed by plotting each k-NN value against the number of communities detected using a “Fast-greedy” clustering algorithm, following which k-NN values ranging from 40 to 60 were deemed the most appropriate based on this analysis (Supplementary Figure 1).

**Table 2:** *F*_ST_ values between pair of populations with *n* > 10, calculated using Weir and Cockerham’s (1984) unbiased approach based on 999 permutations (bottom left matrix)

		1	2	3	4	6	7	8	10	11	12	13	14	15	16	17	18	20	21
1	Magnetic Island		0.04	0.03	0.07	0.06	0.05	0.07	0.10	0.08	0.09	0.13	0.11	0.18	0.17	0.18	0.17	0.18	0.15
2	St Bees Island	0.17		0.03	0.07	0.07	0.05	0.07	0.10	0.07	0.09	0.13	0.11	0.18	0.18	0.18	0.17	0.18	0.15
3	St Lawrence	0.10	0.12		0.05	0.04	0.03	0.04	0.08	0.05	0.07	0.10	0.09	0.15	0.15	0.16	0.15	0.15	0.13
4	Maryborough	0.24	0.26	0.15		0.05	0.04	0.06	0.10	0.07	0.08	0.12	0.11	0.17	0.17	0.18	0.17	0.17	0.15
6	Koala Coast	0.19	0.22	0.12	0.17		0.02	0.03	0.08	0.05	0.07	0.11	0.09	0.16	0.15	0.16	0.15	0.16	0.13
7	Ipswich	0.16	0.19	0.09	0.14	0.06		0.02	0.06	0.04	0.05	0.09	0.08	0.14	0.14	0.15	0.14	0.14	0.12
8	Lismore	0.19	0.21	0.13	0.17	0.10	0.08		0.07	0.05	0.06	0.10	0.08	0.15	0.15	0.16	0.14	0.15	0.13
10	Gunnedah	0.28	0.30	0.21	0.25	0.22	0.20	0.20		0.05	0.04	0.09	0.07	0.13	0.13	0.14	0.13	0.14	0.12
11	Port Macquarie	0.21	0.21	0.14	0.19	0.15	0.12	0.14	0.15		0.04	0.08	0.06	0.13	0.13	0.14	0.13	0.13	0.11
12	Blue Mountains	0.23	0.26	0.16	0.21	0.16	0.13	0.16	0.12	0.11		0.04	0.02	0.07	0.07	0.08	0.07	0.09	0.06
13	Campbelltown	0.33	0.34	0.27	0.32	0.28	0.26	0.26	0.24	0.22	0.15		0.03	0.10	0.10	0.11	0.10	0.12	0.09
14	Southern Highlands	0.28	0.30	0.22	0.27	0.20	0.18	0.21	0.20	0.17	0.06	0.13		0.08	0.08	0.09	0.08	0.10	0.08
15	South Gippsland	0.43	0.46	0.37	0.42	0.34	0.32	0.34	0.35	0.32	0.20	0.33	0.21		0.00	0.01	0.01	0.05	0.02
16	Strzelecki	0.42	0.45	0.36	0.41	0.35	0.33	0.33	0.31	0.30	0.19	0.29	0.23	0.11		0.01	0.01	0.05	0.02
17	French Island	0.49	0.51	0.45	0.47	0.43	0.43	0.43	0.40	0.41	0.32	0.42	0.37	0.34	0.23		0.00	0.04	0.01
18	Cape Otway	0.38	0.40	0.32	0.36	0.30	0.29	0.32	0.29	0.29	0.15	0.28	0.19	0.08	0.09	0.22		0.04	0.01
20	Kangaroo Island	0.53	0.57	0.48	0.50	0.45	0.45	0.47	0.43	0.45	0.34	0.48	0.41	0.39	0.39	0.31	0.25		0.04
21	Mt Lofty	0.39	0.43	0.34	0.39	0.31	0.29	0.31	0.33	0.30	0.20	0.32	0.21	0.06	0.16	0.33	0.08	0.39	

Nei’s unbiased genetic distance (1978) (top right matrix). All values reported were significant to *P* > 0.01

Metrics for populations with *n* < 10 have been removed due to potential subsampling effects of low sample size

Population structuring and proportion of genotypic admixture between populations and regions were also assessed using both a ML approach in Admixture v1.3.0 (Alexander et al. 2009) and a Bayesian approach in Structure v 2.3.4 (Pritchard et al. 2010). Optimal cluster numbers (K) were selected through plotting cross-validation (CV) estimates for Admixture, and through plotting Delta K estimates in Structure. To investigate models of gene flow, isolation by distance (IBD) mantel tests were conducted across the species distribution and for each geographic region in Genalex v6.502 (Peakall and Smouse 2006). In addition, to assess hierarchical levels of population structuring, an analysis of molecular variance (AMOVA) was calculated in Genalex v6.502 fitting geographic regions, populations and individuals as sources of variation (Peakall and Smouse 2006). The groupings for AMOVA evaluations were based on optimum network-based NetView clustering results (Neuditschko et al. 2012; Steinig et al. 2015; Supplementary Table 1, Supplementary Figure 4), and on current subspecies classification and proposed population groupings based on mitochondrial genes outlined in Neaves et al. (2016).

### Phylogenomics

Phylogenetic relationships for all individuals across the species range were inferred using putatively neutral SNP and PAV loci incorporating a ML approach in RAxML v8.2.0 (Stamatakis 2014). In addition, a Bayesian reconstruction method was implemented in MrBayes v3.2.6 (Ronquist et al. 2012) on PAV loci. ML phylogenies were reconstructed using a general time-reversible (GTR) model of nucleotide substitution (ASC_GTRGAMMA) for SNP data, and an optimised site-specific evolutionary rate model (ASC_BINCAT) for PAV loci. For both analyses, a gamma distribution rate for heterogeneity and a “Lewis” method of ascertainment bias correction was applied (--asc-corr). Finally, a rapid bootstrap algorithm (--autoMRE) was implemented for each run to test the support for each of the nodes.

Bayesian phylogenetic analyses were carried out using PAV markers in MrBayes v3.2.6 (Ronquist et al. 2012) and a subset (*n* = 399) of the most informative individuals (based highest call rates, and on observed clustering in ML tree reconstruction) to facilitate convergence of each run. Representative individuals were selected by retaining only a single individual from each minor cluster within the ML tree reconstruction. Bayesian analyses consisted of two runs of 100,000,000 generations each and eight independent chains. Heated chains were set to Temp = 0.10, with a 25% burn-in and a sampling frequency of 1000. Dirichlet prior states were set to 48:52, which were calculated based on observed frequencies of absence (“0”) and presence (“1”). Runs were completed if standard deviations between runs were below 0.05, and were independently assessed for convergence using Tracer v1.6 (Rambaut et al. 2014). In addition to consensus trees produced by both RAxML and MrBayes, maximum credibility consensus trees were constructed using TreeAnnotator v1.7.0 (Rambaut and Drummond 2013), with a burn-in of 10% and a posterior probability cut-off of 25%. Each consensus tree was then constructed using a NJ approach, and visualised and edited in FigTree v1.4.2 (Rambaut and Drummond 2012). All individuals were used to create an initial tree, however, for clarity, a subset of only the most distinct individuals was used for construction of the final consensus tree for each statistical method.

## Results

### SNP calling and quality control

A total of 15,004,234 sequence reads, corresponding to 19,187 polymorphic loci were obtained across 800 individuals from Diversity Arrays. Following genotype filtering, 35 individuals were removed from the dataset due to poor SNP coverage and 13,818 SNPs (72%) were removed from the dataset for violating filtering parameters, with low call rate being the primary factor. A total of 104 sex-linked markers (X chromosome = 86, Y chromosome = 18) were identified and removed from the working dataset, and the remaining dataset of 5265 SNPs was then tested for conformity to HWE (three SNPs removed) and LD (659 SNPs removed). A final set of 4606 unique autosomal SNPs were retained for downstream analysis. A total of 22,022 PAV markers were initially identified across all individuals. Following filtering, a total of 6102 PAV markers were retained for use in phylogenetic reconstructions.

### Identification of signatures of selection

Weak to moderate signatures of selection were identified among populations investigated at both predefined FDR levels (FDR = 0.01 and 0.001). A total of 137 SNPs (100 directional and 37 purifying/balancing) were identified at FDR = 0.01, and 71 were identified at FDR = 0.001. Average *F*_ST_ across populations calculated using neutral loci was 0.18 (SD ± 0.06), with average *F*_ST_ for directional outliers being markedly higher (average *F*_ST_ = 0.37, SD ± 0.17). When NJ trees were constructed based on 1-proportion of shared alleles genetic distances for each locus type, branch lengths were slightly longer and more uniform among individuals using neutral SNPs when compared with directional outlier SNPs (Fig. 5). However, the directional SNPs displayed very similar clustering patterns across all populations, with no single population (or bioregion) showing signatures of extreme selection. When estimating the magnitude of population differentiation within and among bioregions due to selection alone, the average within-bioregion *F*_ST_ was very low at 0.04 (SD ± 0.03) and average between-bioregion *F*_ST_ was 0.20 (SD ± 0.11). Between-bioregion *F*_ST_ differences increased according to bioregion differences in a clinal pattern, whereby neighbouring bioregions displayed an intermediate average *F*_ST_ difference of 0.13 (SD ± 0.08), by comparison the greatest difference of 0.39 was observed between the most divergent bioregions (Sydney Basin (SYB) versus Brigalow Belt North (BBN); Table 1).

No putatively identified outlier loci were associated with annotated genic regions, and when flanking regions for each SNP were compared with existing genomic databases, only 20 markers fell within ±2000 bp of annotated gene region, with a matching identity and coverage of 100% (Supplementary Table 4). However, it could not be confirmed that these were definitively linked to functional regions.

### Population-specific and regional genetic diversity

Average observed heterozygosity (Ho) across populations ranged from 0.09 to 0.18, and average expected heterozygosity (He^corr^) ranged from 0.10 to 0.20 (Table 1). Among populations, the Blue Mountains population displayed the highest heterozygosity values (He^corr^ = 0.20), whereas the French Island population showed the lowest levels (He^corr^ = 0.10). Percentage of polymorphic loci ranged from 37.7% (South Gippsland) to 82.5% (Campbelltown) (Table 1). *F*_IS_ values were generally close to zero, with an average of 0.04, and ranged from −0.03 (St Bees Island) to 0.19 (Kangaroo Island). Average sMLH was highest in Port Macquarie (1.21), whereas Kangaroo Island displayed the lowest level (0.58). Frequencies of rare alleles within populations ranged from 0.04 to 0.18 across the species range, with Port Macquarie and Blue Mountains populations having the highest levels (Table 1). When comparing regions based on phylogenetic clades (north versus south, see below), sMLH, %PL, Ar and Ap values were higher in populations residing in the northern region (Table 1 and Fig. 1).

### Broad-scale population structuring

**Fig. 2:**
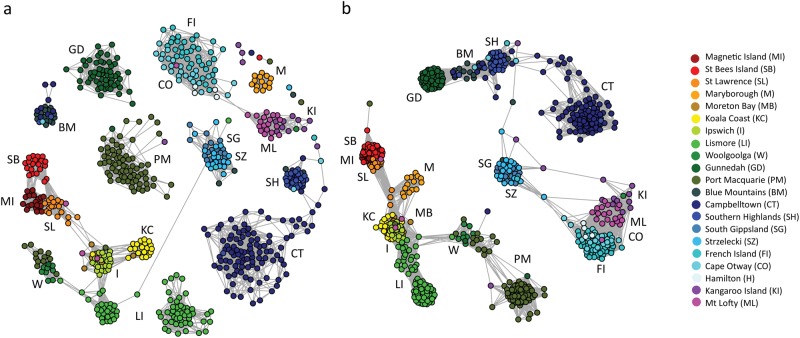
Netview R clusters at multiple k-NN values, **a** k-NN30, **b** k-NN60

Across the sampling range, two clear genetic clusters were identified through Netview R clusters at k-NN ≥ 40, with major regional clusters being observed at k-NN = 30 (Fig. 2). The divide between the two overarching clusters was observed within the Blue Mountains population. Most Victorian and South Australian populations clustered closely together, with the exception of South Gippsland and Strzelecki sourced samples, which were distinct from the other southern populations, but indistinguishable from one another. The majority of individuals sampled from the same location clustered tightly to their predefined populations, indicating that designated populations were appropriate (Fig. 2). Populations within a specific bioregion (IBRA version 7, 2012) also tended to cluster more closely together at all predefined k-NN clustering levels.

**Fig. 3:**
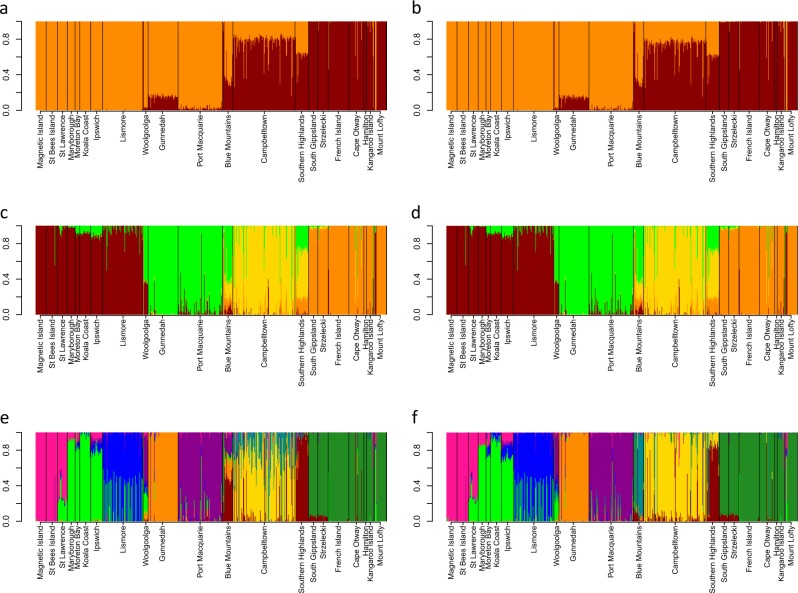
Proportion of genotypic admixture between regions calculated using a maximum likelihood approach in Admixture v1.3.0 (Alexander et al. 2009), and a Bayesian approach in Structure v2.3.4 (Pritchard et al. 2010); **a** *K* = 2 (Admixture), **b** *K* = 2 (Structure), **c** *K* = 4 (Admixture), **d** *K* = 4 (Structure), **e** *K* = 9 (Admixture), **f** *K* = 9 (Structure)

Both ML and Bayesian approaches (Admixture and Structure, respectively) returned the same results at each respective K value (Figs. 3a–f). Each analysis method detected different levels of differentiation, and so returned different optimal cluster number despite returning the same structuring patterns. For the Admixture analysis, *K* > 9 was optimal (Fig. 3e, Supplementary Figure 4a), with genetic admixture highest within the Blue Mountains and Campbelltown regions, which was consistent with the clustering patterns observed in Netview R at lower k-NN values. Structure results indicated that the most likely number of clusters was *K* = 4 (Fig. 3d, Supplementary Figure 4b). These clustering patterns (*K* = 4) were similar to those observed in Netview R plots at kNN 40–50 (Supplementary Figure 3), and in the subclades observed in phylogenetic analysis (see below). When *K* = 2 was visualised for comparison with the two overarching clusters observed in Netview R plots (kNN > 60) and phylogenetic trees (see below), admixture can be observed throughout NSW populations (with exception of the Lismore population), with higher levels of admixture observed within the Sydney region (Campbelltown and Southern Highland populations; Figs. 3a–f).

**Fig. 4:**
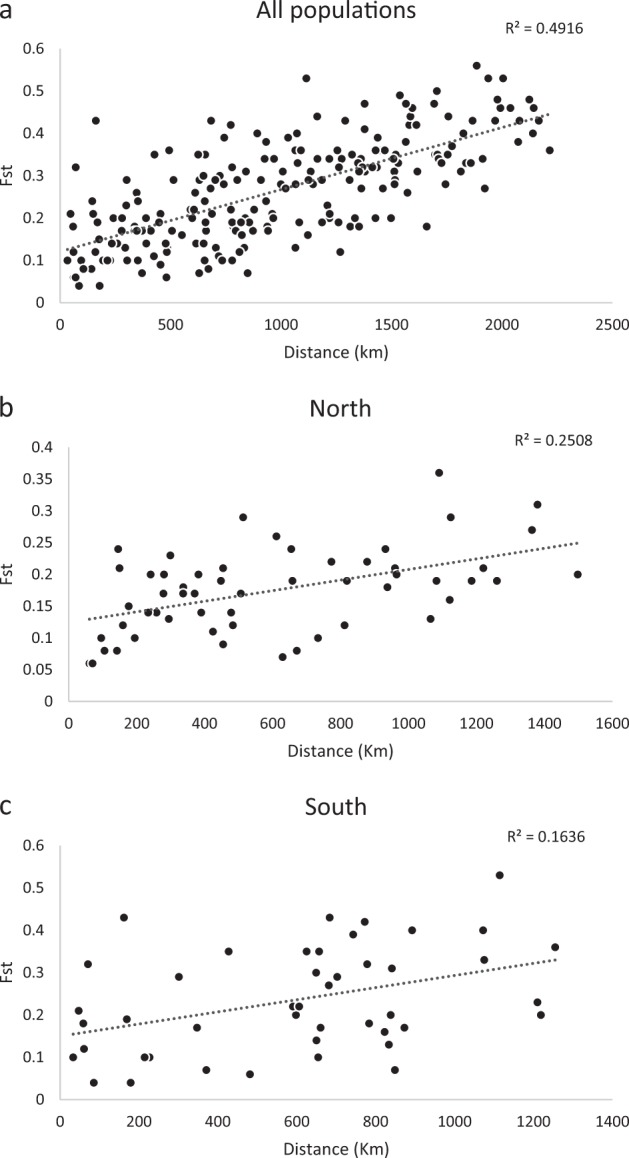
Mantel tests to investigate an isolation by distance model for gene flow between populations and regions, **a** all populations, **b** Northern populations, **c** Southern populations

The results of the IBD Mantel test revealed a moderate to strong correlation with genetic distance and geographical distance when evaluating all populations across the species range (*R*^2^ = 0.49; Fig. 4a). When north and south regions were analysed separately, based on Netview R clustering, populations from both regions showed positive relationships between genetic and geographical distance, although less strong for the southern region (Figs. 4b, c).

Pairwise *F*_ST_ values were highly variable ranging from 0.04 between the geographically close Blue Mountains and Southern Highlands in NSW, to 0.56 between St Bees Island and Mt Lofty, which are close to the geographical ends of the species range (Table 2 and Fig. 1). Populations with documented genetic bottlenecks displayed higher average pairwise *F*_ST_ values (i.e., French Island AvF_ST_ = 0.36 ± 0.08, Mount Lofty AvF_ST_ = 0.42 ± 0.09, Kangaroo Island AvF_ST_ = 0.3 ±0.12) compared with populations with more stable population histories (i.e., Blue Mountains AvF_ST_ = 0.15 ± 0.06). This indicated that these genetic bottlenecks may have skewed or inflated *F*_ST_ values (Pearse and Crandall 2004). The high level of genetic divergence observed between populations at opposite ends of the species distribution supported an IBD dispersal model for this species (Fig. 4). Average pairwise *F*_ST_ across all populations was 0.27 (SD ± 0.12), and overall average within the northern group (based on Netview R clustering) was lower than in the southern groups (0.17, SD ± 0.06 and 0.27, SD ± 0.12, respectively). Partitioning of genetic variance based on AMOVA tests revealed that among individuals and within individuals accounted for most of the genetic variation (~30% and ~40%, respectively) independent of the groupings applied to populations. Among-population variance ranged from 14.3% to 20.4% between analyses, reflecting moderate population differences corresponding to other analyses. Among groups as the source accounted for the least amount of genetic variance, with the largest variance of 13.6% recorded when populations were grouped together based on genetic similarities and optimum number of clusters (i.e., *F*_ST_ and Netview R data; Table 2 and Fig. 2a), whereas the minimum of 8.4% was obtained when populations were grouped based on Neaves et al. (2016) proposed groups. Groups based on northern and southern regions (as defined by Netview R analysis; Fig. 2b), accounted for 10.5% of the genetic variance, and while groups based on current subspecies classification described 13.0% of the variation (Supplementary Table 1).

### Phylogenomics

Both ML and Bayesian phylogenetic reconstruction, for both SNP and PAV markers revealed overall very similar topologies and node support with two major clades separating at the Blue Mountains/Campbelltown population in NSW (Fig. 6; Supplementary Figure 2a, b). All populations north of the Blue Mountains (including the majority of Blue Mountains sourced individuals) clustered together in a single overarching northern group and all populations below Campbelltown (including the majority of Campbelltown individuals) clustered together in a southern clade (Fig. 6; Supplementary Figure 2a, b). Individuals were generally placed within their assigned populations/regions with the exception of individuals from Blue Mountains and Campbelltown, which had individuals in both the northern and southern clades. This was again consistent with Netview R clustering (Fig. 2). Branch lengths were generally shorter among Victorian and South Australian populations, with the PAV trees placing all Victorian and South Australian samples as a subset clustering off the southern NSW populations (specifically the Southern Highlands group). Strong bootstrap support and posterior probability (>0.8) was observed for both major clades (north and south) in all reconstruction methods, with variable support at the intermediate nodes at a population level (0.42–1.0). A high degree of population mixing was observed within the southern clade, where individuals assigned to one population clustered with other populations (often a neighbouring population). Populations within VIC and SA, with the exception of South Gippsland and Strzelecki, were intermixed in all phylogenetic tree reconstructions. South Gippsland and Strzelecki appear to be relatively divergent from the other southern koala populations, forming a distinct subclade apart from other southern populations in both phylogenetic constructions using PAV markers (Figs. 6a, c).

## Discussion

Using a species-wide sampling strategy across heterogeneous environments, and high-density genome-wide markers, here we show that remnant koala populations display comparable levels of diversity to that of many other wild species (Kjeldsen et al. 2016), and that their broad-scale population differentiation is primarily driven by an IBD genetic structure model (49% of genetic variance) with clinal local adaptation. Detailed genetic structure patterns closely reflect population gene flow based on geographical locations, barriers to dispersal and documented translocations. Hierarchical genetic clustering analysis revealed two shallow overarching genetic groups present across Australia with genetic admixture, which is indicative of a weak historical genetic division within the Sydney/Blue Mountains region. When assessing signatures of selection, the results of this study indicate that populations within bioregions are experiencing very similar selective pressures, whereas different selective forces are acting on bioregions in a clinal pattern across the koala’s east coast Australian distribution. Specialist species are expected to be more sensitive to selective pressures and stochastic change in the environment (Franzén et al. 2012; Kierepka et al. 2016). However, our results indicate that the majority of koala populations are comparable in the levels of genetic diversity and substructuring (Table 2; Fig. 2) to many other outbred species (Kjeldsen et al. 2016), except for those populations that have been impacted by population bottlenecks and/or translocations. The pattern of population structuring observed across Australia can be largely attributed to an IBD dispersal model, and limited local adaptation within bioregions. The results of this study suggest that although the koala is a widely considered to be a specialist folivore (Adams‐Hosking et al. 2012; Hume 1982), this dietary specialisation has not limited the koala’s ability to maintain gene flow and locally adapt across divergent environments. As long as specific diet requirements are met (Moore et al. 2005), they appear to behave like a generalist species with no specialised environmental requirements to suit a specific bioregion, beyond the requirement for the presence of appropriate eucalypt species. These genetic patterns can also be observed in the phylogenetic and AMOVA analysis presented here. These data indicate that at a taxonomic level, koalas belong to a single genetic group with the majority of genetic variation being between individuals, and as such does not support the three current subspecies classification.

### Species-wide genetic divergence and signatures of selection

Previous koala phylogenetic studies have revealed between two and four genetic “clades” across Australia using mtDNA sequence data (Houlden et al. 1999; Neaves et al. 2016). Although mtDNA has been traditionally used for phylogenetic reconstruction across many species, it may not be ideal for resolving phylogenetic relationships in koala. The reported low level of koala mtDNA gene diversity (Houlden et al. 1999; Neaves et al. 2016) and absence of a suitable molecular clock (Neaves et al. 2016) limits its use in generating highly informative phylogenetic data with robust clade support. The limited resolution provided by mtDNA markers do not allow for a holistic assessment of the koala’s ability to adapt to changes in the environment. Genome-wide markers provide an added level of insight into the genomic structure and phylogenetic history of koala populations by sampling both adaptive and neutral regions of the genome (Kirk and Freeland 2011). Both phylogenetic analysis (Fig. 6) and hierarchical clustering methods (Figs. 2 and 3) utilised in this study reveal two historical shallow genetic groupings or “clades” across Australia. Although these groups returned relatively weak signals, when compared with current regional structuring, the two genetic groups still have strong support of separation within the Sydney Basin, splitting the Blue Mountains and Campbelltown populations (Fig. 1) between the northern and southern clades in all phylogenetic reconstructions. Despite this clear separation at one point in the koala’s evolutionary history, admixture is also present within this region, indicating that even though historical barriers to gene flow have been present for a period of time, gene flow between northern and southern clades is currently occurring (Fig. 3).

Although difficult to confirm the specific barrier, based on the location of this genetic division, it is possible that the “Hunter Valley rift” contributed to this divergence. In studies on other species, including ancient assassin spiders (Rix and Harvey 2012), garden skinks (Chapple et al. 2011), giant burrowing frogs (Penman et al. 2005), common froglets (Symula et al. 2008), eastern yellow robins (Pavlova et al. 2013) and brush-tailed rock wallabies (Hazlitt et al. 2014), the Hunter Valley rift has been implicated in driving the speciation of several other species groups (during the mid-late Miocene era). Given the geological history within this region involving dramatic changes in landscape and vegetation structure (Byrne 2008; Dubey et al. 2010), it is possible for this historical barrier to have previously restricted movement of koalas. Furthermore, habitat type and terrain could also have played a role in koala divergence, with the Great Dividing Range falling within the region of admixture observed in this study. Significant climatic variation during the mid-Pleistocene (Byrne 2008), causing shifts from warm and wet conditions, to cool and dry conditions, leading to habitat expansions and contractions (Dubey et al. 2010), may also have contributed to koala divergence. Interestingly, no significant divergence was observed when mtDNA markers were used for phylogenetic reconstruction (Neaves et al. 2016). The discrepancy between reconstructions may be a result of a number of factors, not the least of which being that two different genetic marker types, with different mutation rates, were utilised between these studies. Despite this, it is evident based upon the presence of clear genetic admixture (Figs. 3a, b) that the historical barrier is no longer significantly affecting gene flow in present day populations.

The current study strengthens support for rejection of any subspecies classification in the koala (Houlden et al. 1999; Kjeldsen et al. 2016), with no evidence observed here for the originally described three distinct subspecies (Thomas 1923; Troughton 1935, 1941), which were based largely on state legislative borders and morphological differences. There was no indication, in any of our analyses that three distinct evolutionary significant units (ESUs) are present based on previous classifications. Hierarchical AMOVAs indicated that among and within-individual sources of variation accounted for most of the genetic variance (approximately 30% and 40% respectively; Supplementary Table 1). This indicates that most of the genetic variation and evolutionary potential within the species is observed at the individual level rather than in geographical regions or populations. Variation among groups accounted for the smallest proportion of the genetic variance (8.4–13.7%) supporting only a single ESU, rejecting subspecies classifications and other groupings based on phylogenetic relationships (e.g., Fraser and Bernatchez 2001; Neaves et al. 2016 and Fig. 6).

There is no doubt that conservation of evolutionary processes and ecological viability of koalas is of fundamental importance. Based on adaptive divergence and population connectivity data, we propose that koalas should be classified under a single ESU. First, based on koala distribution data (Fig. 1), there is no obvious geographic delineation of koalas into distinct groups with significant historical isolation. Second, contemporary reproductive isolation is not observed, with moderate gene flow observed between proximal populations throughout their distribution (Table 2). Mantel test results indicate that an IBD population structuring effect is evident across the species range and explains a large proportion of the genetic variance observed (*R*^2^ = 49.2%, *p* < 0.001). Although a historical north/south separation is observed within the Sydney Basin (Fig. 6), the divergence between the two clades is small, based on short basal branch lengths, relative to tip lengths and contemporary admixture is high between regions; up to 50% between neighbouring populations within the Sydney Basin region (Figs. 3a, b). Finally, koala morphological traits and genetic signatures of selection follow a clinal pattern across an environmental gradient rather than distinct groupings (Martin and Handasyde 1999; Supplementary Table 3).

**Fig. 5:**
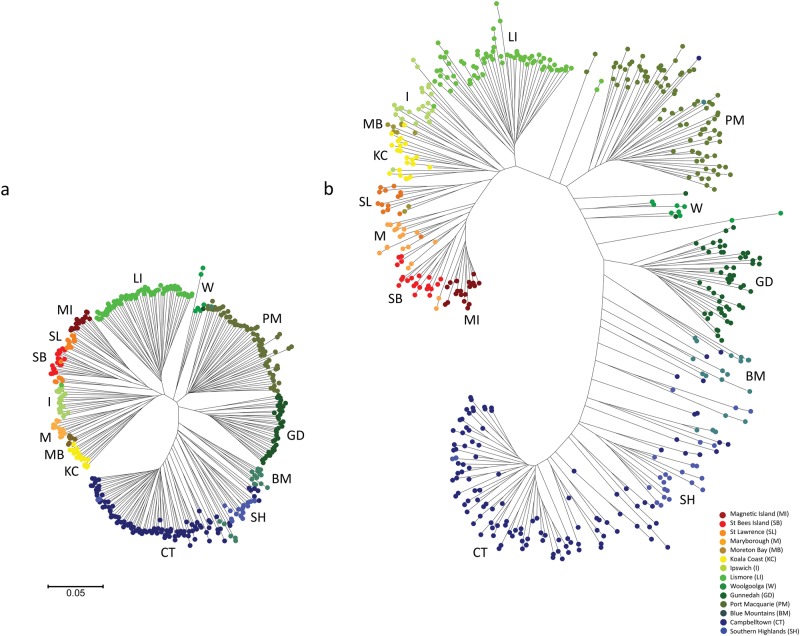
Genetic distance (1-Proportion of shared alleles) calculated based on **a** neutral and **b** putatively identified SNPs under selective pressures, trees constructed using a neighbour-joining approach in MEGA6 (Tamura et al. 2013)

The clinal phenotypic variation observed in the koala across its distribution is not surprising, based on the large species range (from wet tropics in Northern Australia through to temperate climates in the Southern Australia) (Briscoe et al. 2015). Phenotypic variation has been observed in a number of other species across environmental gradients, including red deer (*Cervus elaphus*) (Post et al. 1997), *Bicyclus* butterflies (*Bicyclus sp*.) (Brakefield and Reitsma 1991), red squirrels (*Sciurus vulgaris*) (Réale et al. 2003) and even humans (*Homo sapiens*) (Campbell and Tishkoff 2010; Manica et al. 2007). Much of this variation is attributed to adaptation to different climates and habitats (Manica et al. 2007; Briscoe et al. 2015). Variable ecological pressures can result in different selective pressures across different bioregions (Gienapp et al. 2008). Overall climate and habitat type vary significantly from the northernmost regions of Australia, through to the southern regions, with large differences in temperature ranges, rainfall, soil types and vegetation structure (Hughes 2003). Interestingly, despite this variation in climate and habitat across the koala’s range, no strong signatures of selection were identified in any specific bioregions or population (Fig. 5). The largest differences observed were between bioregions, with more variation observed between bioregions, as opposed to within a bioregion, regardless of geographic distance (Supplementary Table 3). This suggests that the koala is capable of inhabiting and adapting to a broad variety of environmental conditions as long as suitable dietary components are available.

### Population genetic diversity and substructuring

**Fig. 6:**
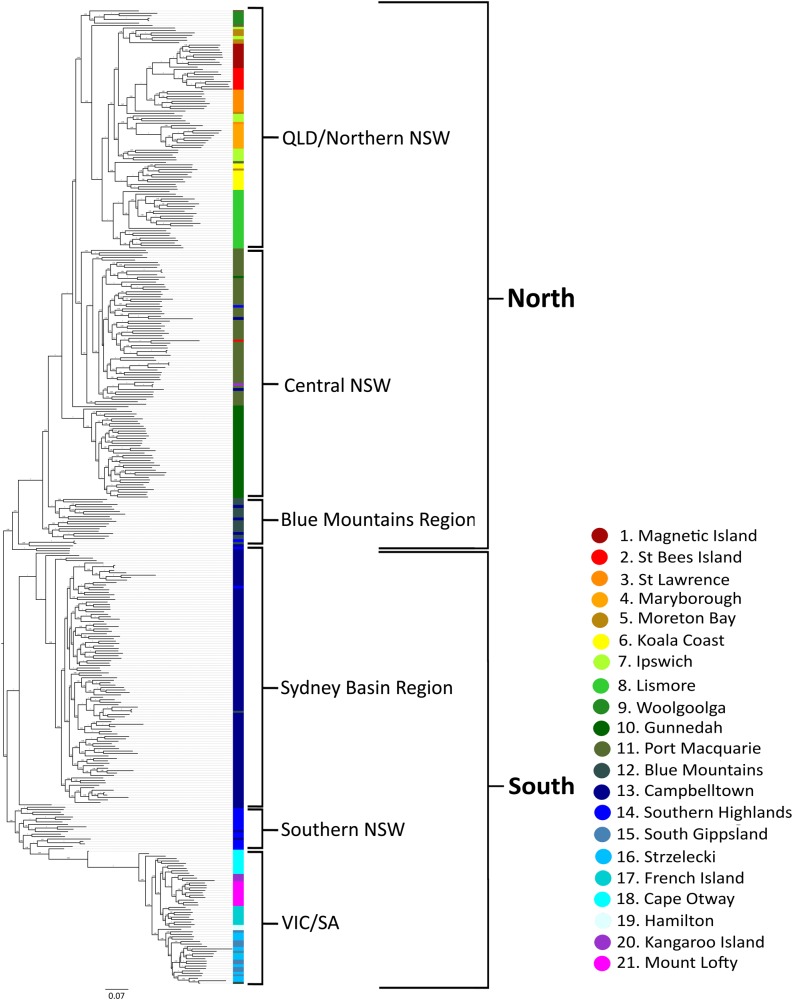
Phylogenetic reconstruction using a subset of 399 representative individuals. Tree constructed using a maximum likelihood approach based on PAV markers

Ecological history appears to have a direct effect on contemporary genetic diversity levels, with all southern populations (populations sampled from VIC and SA) displaying clear reductions of diversity (Table 1). Historic records of hunting and subsequent reintroductions (Menkhorst 2008; Wedrowicz et al. 2018) from island populations with a small number of founders are likely to have led to the low diversity levels seen in this region. Hunting in the early 20th century decimated many Victorian mainland populations (estimated <1000 animals left by 1930) and led to the complete extinction of South Australian populations (Menkhorst 2008). This is reflected in the lack of distinct population groupings seen in most mainland Victorian populations (Fig. 6). Despite active translocations becoming less frequent, only approximately 16 generations (within approximately 100 years, based on a 6-year generation interval) separate the initial translocations from Victorian island populations back to the mainland, with several additional translocation events within this time. With so few generations, with active translocations, it is perhaps not surprising that there was so little differentiation observed across this region. Some animals within the Strzelecki and South Gippsland regions are said to have escaped hunting (Menkhorst 2008), and the current study indicates that these populations are distinct from the rest of the Southern populations (Figs. 2 and 5), although overall diversity was still relatively low (He^corr^ = 0.11 and 0.11, respectively). This pattern was also observed in a recent study using microsatellite markers, where the South Gippsland region was observed to be distinct from other Southern populations (Wedrowicz et al. 2018). Interestingly, despite being from the same region (Lee et al. 2011), there were also low levels of differentiation observed between the samples obtained from the Strzelecki Ranges and the remainder of the South Gippsland samples (*F*_ST_ = 0.11). Animals that persisted in this region may have carried remnant diversity, and genotypes, which were lost elsewhere in southern regions as a result of widespread hunting, may have been retained in these populations.

Interestingly, population reductions do not appear to have adversely effected the koala’s ability to thrive in the short term, as population numbers are increasing in several areas (e.g., Kangaroo Island and French Island). Genetic relationship patterns clearly show that most mainland Victorian koalas are very similar to those from French island (Figs. 2 and 3), but the high *F*_ST_ values in this region are likely inflated due to repeated genetic bottlenecks and random genetic drift effects (Pearse and Crandall 2004). Variation in vegetation structure across the east coast of Australia may also affect abundance of animals across the species range (Davies et al. 2013; Dudaniec et al. 2013), although overall abundance within a region appears to be a poor indicator of genetic variation in this case.

Of the regions sampled in this study, groups of populations that were sampled from areas surrounding protected habitats (e.g., the Blue Mountains region) appeared to have higher levels of admixture, and generally higher diversity values than those surrounding suburban areas (Fig. 3; Table 1). The higher levels of diversity in regions surrounding protected habitat may have been maintained by the increased genetic connectivity between these populations (Figs. 2 and 3), reducing the effects of genetic drift, which can lead to random loss of alleles in smaller, isolated populations (Allendorf 1986). The dietary specialisation of koalas also restricts them to areas that can support their primary food tree species, and fragmentation of this habitat by either changes in climate, or through human activities can restrict animal movement, thus reducing overall connectivity (Devictor et al. 2008). In areas of continuous, favourable habitat, successful dispersal and subsequent settlement of juvenile koalas (both male and female) is greater than in urban areas with fragmented habitat. This successful dispersal and settlement is largely attributed to a lower rate of juvenile deaths by dog attacks and car collisions (Lassau et al. 2008; Tucker et al. 2007). If dispersal patterns and social dynamics of koala populations vary across the range based on overall habitat structure, this is likely to have an effect on genetic structure between regions.

A number of island populations of koala across Australia were sourced from a limited number of founder individuals (Menkhorst 2008). Given the dietary specialisation of the koala, isolated island populations are further at risk of changes to local habitat and stochastic events. The two northern island populations sampled here displayed diversity levels comparable with mainland populations (Table 1), and returned *F*_IS_ values close to zero, which is consistent with outbred populations (Magnetic Island = 0.01, and St Bees Island = −0.03). Despite the small number of animals that colonised these populations, (Magnetic Island ≥ 18 wild individuals, Martin and Handasyde 1999; St Bees Island ~ 12–17 wild individuals, Lee et al. 2012), there was no evidence of a founder effect or reduced genetic diversity when compared to the mainland. Furthermore, both St Bees Island and Magnetic Island contain large areas of relatively unmodified habitat, and few introduced predators, as a result of limited anthropogenic interference (Pfeiffer et al. 2005), and this may have contributed to a relatively swift colonisation of islands that would reduce the effect of genetic drift and loss of diversity (Zenger et al. 2002). In comparison, the two southern introduced island populations (French Island founded by ~ 2–3 wild individuals; Kangaroo Island founded by ~18 individuals from French Island, Masters et al. 2004) returned positive *F*_IS_ values (French Island = 0.09 and Kangaroo Island = 0.19). The French Island population has been used repeatedly to supplement mainland Victorian and South Australian populations. These translocations are concerning as French Island displays one of the lowest heterozygosity values across Australia (Ho = 0.11), and the high *F*_IS_ values observed here should also be noted, as any further translocations from this island may impact diversity levels in the target population, particularly if the target population was originally sourced from French Island. Similarly, the Kangaroo Island population is of particular concern, as in addition to it returning the highest inbreeding value, it also has the lowest observed heterozygosity value (Ho = 0.09), lowest average standardised individual multilocus heterozygosity (sMLH = 0.59) and the highest IR value (IR = 0.83). These populations have been highlighted as having reduced genetic diversity in the past (Cristescu et al. 2012; Lee et al. 2012b; Taylor et al. 1994), with reported cases of physical abnormalities being present in these populations, which is often a result of higher rates of inbreeding (Cristescu et al. 2012). This study further confirms the need for careful management of these populations to avoid further loss of diversity. When evaluating mainland populations, inbreeding coefficients were generally close to zero, with the exception of Lismore (*F*_IS_ = 0.11), and the Blue Mountains (*F*_IS_ = 0.1) that returned positive *F*_IS_ values. However, Netview R clustering indicates that population substructuring is present in these populations (Fig. 2), and with relatively high diversity levels, these *F*_IS_ values are a result of Wahlund effect rather than inbreeding (Christiansen 1988; Sinnock 1975).

The Blue Mountains population appears to hold much of the genetic diversity of the species, with a large proportion of rare alleles being present in the Blue Mountains animals (see also Lee et al. 2010; Table 1). This is important, as other regions were previously highlighted as key populations for research and conservation to conserve overall species diversity (i.e., South Eastern QLD; Fowler et al. 2000; Lee et al. 2012; Ruiz-Rodriguez et al. 2014; Wilmer et al. 1993), because they were said to have escaped hunting, and thus maintain remnant historic diversity (Cocciolone and Timms 1992; Fowler et al. 2000; Lee et al. 2010, 2012; Ruiz-Rodriguez et al. 2014; Wilmer et al. 1993). Although this may be true to an extent, the Blue Mountains regions (and other areas within NSW) appear to have higher diversity levels, and rare genetic variants (Table 1). Subsequently, although it is important to preserve all populations of koala, this region should be highlighted for future study if we are seeking to preserve existing diversity for the entire species. Southern populations appear to be less diverse as a whole, and this is likely a result of genetic bottlenecks, translocations and reintroductions in the past 100–200 years. Comparisons of overall species diversity can be difficult to accurately estimate, and differences are seen between studies depending on the genetic marker type used, which populations are sampled, and which statistical methods are employed to filter genetic data or estimate diversity (Fowler et al. 1998a, b; Houlden et al. 1999; Kjeldsen et al. 2016; Lee et al. 2010a 2012; Neaves et al. 2016; Ruiz-Rodriguez et al. 2014). Nonetheless, based on data from this study and Kjeldsen et al. (2016), many koala populations display levels of genome-wide genetic diversity that are comparable to other outbred animal populations with similar life histories. Furthermore, across koala genetic studies to date, general trends of lower diversity in Victorian and South Australian populations, and higher levels of diversity within NSW and QLD populations, have been widely observed (Cocciolone and Timms 1992; Cristescu et al. 2012; Fowler et al. 2000; Houlden et al. 1996; Kjeldsen et al. 2016; Lau et al. 2014; Lee et al. 2010a 2012a; Neaves et al. 2016; Taylor et al. 1994; Tsangaras et al. 2012; Wilmer et al. 1993).

### Management recommendations and conclusions

Management of species with specialised ecological requirements can be a challenge, given their inherent sensitivity to changes in habitat structure. Classification into appropriate ESUs is crucial for maximising the evolutionary potential of a species-group, particularly when environmental change threatens the species as a whole. Taxonomic uncertainty can complicate conservation management resulting in potential mixing of different species (or subspecies), which in extreme scenarios can lead to outbreeding depression (Frankham 2003). From a legislative standpoint, legal protection is often defined based on major species groupings, or ESUs (Funk et al. 2012), and so resolving these groups accurately is essential to conservation efforts. In the current study, two shallowly divergent phylogenetic clades were observed (Fig. 6). However, high levels of genetic admixture observed between these clades, particularly at their geographic interface (Sydney Basin region, NSW), and a clear clinal relationship between genetic divergence and geographic location (accounting for 49% of genetic variance; Fig. 4a), were observed. Furthermore, on a hierarchical level ~70% of the total genetic variance is observed at the individual level, with <13% for the subspecies classifications still recognised by many government and non-government organisations (i.e., those described in Thomas 1923 and Troughton 1935, 1941). These results indicate that for the koala, only a single ESU is present, which is in keeping with the most recent mitochondrial research (Neaves et al. 2016).

Currently koala populations are managed based on arbitrary geographic distances, with translocations and movement of animals often restricted to local government boundaries, or prohibited completely (Queensland Parks and Wildlife Service 2006; NSW National Parks and Wildlife Service 2001; National Parks South Australia 2016). This management regime is variable across the species range, and perhaps not always ideal to maintain natural genetic structuring. This study indicates that any active management of koalas needs to be considered at a regional level, likely corresponding to environmental bioregions. Future management strategies would need to be considered on a case-by-case basis for each region, because ecological histories vary significantly across the range. Although no specific regions were identified as showing extreme signatures of local adaptation, much of the selective differentiation observed was accounted for between these bioregions. Similarly, the strong IBD effect observed in this study indicates that, although a standard arbitrary distance may not be appropriate, geographic distance between populations should be considered, particularly if managing across bioregions.

Across these genetic groups/bioregions, populations containing particularly high levels of genetic variation and diversity should be highlighted in future management plans (e.g., Sydney Basin region – Blue Mountains population). These populations could be considered to be reservoirs holding substantial species diversity. However, the effects of local adaptation between bioregions should not be ignored, because movement of animals into unsuitable habitats may result in overall reduced fitness (Frankham 2003). Despite some regions containing higher levels of diversity, it should be noted that even within a single bioregion, the majority of genetic variance is still accounted for at an individual level, rather than within populations or groups. This variation highlights the importance of conserving koala populations wherever possible. This study gives the most comprehensive genome-wide assessment of koalas, and provides vital information for the informed management of these animals across their range.

### Data archiving

Data available from the Dryad Digital Repository: https://datadryad.org//resource/doi:10.5061/dryad.1683r1s

## Electronic supplementary material

Supplemental material - information

Supplementary Figure 1

Supplementary Figure 2. a)

Supplementary Figure 2. b)

Supplementary Figure 3

Supplementary Figure 4

Supplementary Table 1

Supplementary Table 2

Supplementary Table 3

Supplementary Table 4
