# Supplementary material for: Genomic comparisons reveal biogeographic and anthropogenic impacts in the koala (Phascolarctos cinereus): a dietary-specialist species distributed across heterogeneous environments
Source: Heredity (Edinb). 2018 Sep 12;122(5):525–44. doi: 10.1038/s41437-018-0144-4 (PMC6461856; doi:10.1038/s41437-018-0144-4)
Supplement: Supplementary file 1 — Supplemental material - information [file 41437_2018_144_MOESM1_ESM.pdf]

Supplementary Figure 1. Netview R k-NN selection plot based on three clustering algorithms.

Supplementary Figure 2. a) Maximum likelihood trees constructed using SNP markers, **b)** Bayesian tree constructed using PAV markers

Supplementary Figure 3. Netview R clusters at intervals of k-NN=10, from k-NN=10-60

Supplementary Figure 4. K plot selection for Admixture v1.3.0 (Alexander *et al.* 2009) and Structure v2.3.4 (Pritchard *et al.* 2000) results; **a)** CV plot of Admixture results, **b)** Delta K plot of Structure results

Supplementary Table 1. Hierarchical analysis of molecular variances between multiple groupings of populations based on Netview R clustering at various k-NN values. **a)** Northern clade and Southern clade, **b)** Four Groupings (1-[Magnetic Island, St Bees Island, St Lawrence, Maryborough, Moreton Bay, Koala Coast, Ipswich, Lismore], 2- [Woolgoolga, Port Macquarie], 3-[Gunnedah, Blue Mountains, Campbelltown, Southern Highlands], 4- [South Gippsland, Strzelecki, French Island, Cape Otway, Hamilton, Mt Lofty, Kangaroo Island]), **c)** Five Groups (based on mtDNA genetic divides proposed by Neaves *et al.*2016), **d)** Ten groups (1-[Magnetic Island, St Bees Island, St Lawrence, 2- [Maryborough], 3- [Moreton Bay, Koala Coast, Ipswich], 4- [Lismore], 5- [Woolgoolga, Port Macquarie], 6-[Gunnedah], 7- [Blue Mountains, Campbelltown, Southern Highlands], 8- [South Gippsland, Strzelecki], 9- [French Island, Cape Otway, Hamilton], 10- [Mt Lofty, Kangaroo Island])

Supplementary Table 2. F_{ST} values between pair of populations with n>10, calculated using Meirman's approach (2004) based on 999 permutations (bottom left matrix)

Supplementary Table 3. F_{ST} values for putatively neutral and directional outliers, and comparisons to bioregions.

Supplementary Table 4. Basic Local Alignment Search (BLAST) results for putatively identified outlier loci, including a ±2000 bp flanking region surrounding each identified SNP.
