# Supplementary figures and images for: Genomic comparisons reveal biogeographic and anthropogenic impacts in the koala (Phascolarctos cinereus): a dietary-specialist species distributed across heterogeneous environments
Source: Heredity (Edinb). 2018 Sep 12;122(5):525–44. doi: 10.1038/s41437-018-0144-4 (PMC6461856; doi:10.1038/s41437-018-0144-4)

Communities in mkNNGs

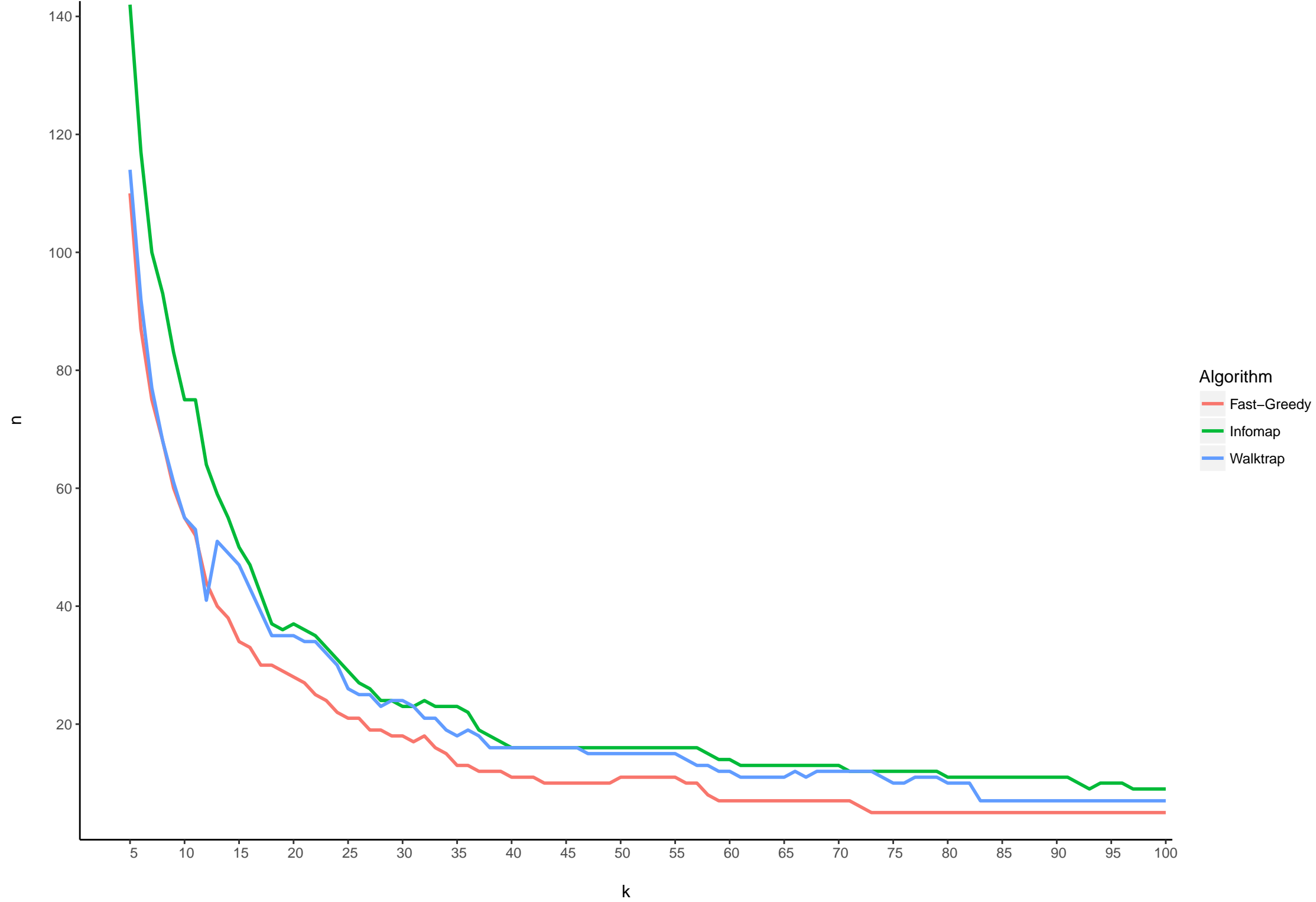

Supplement: Supplementary file 2 — Supplementary Figure 1 [file 41437_2018_144_MOESM2_ESM.pdf]

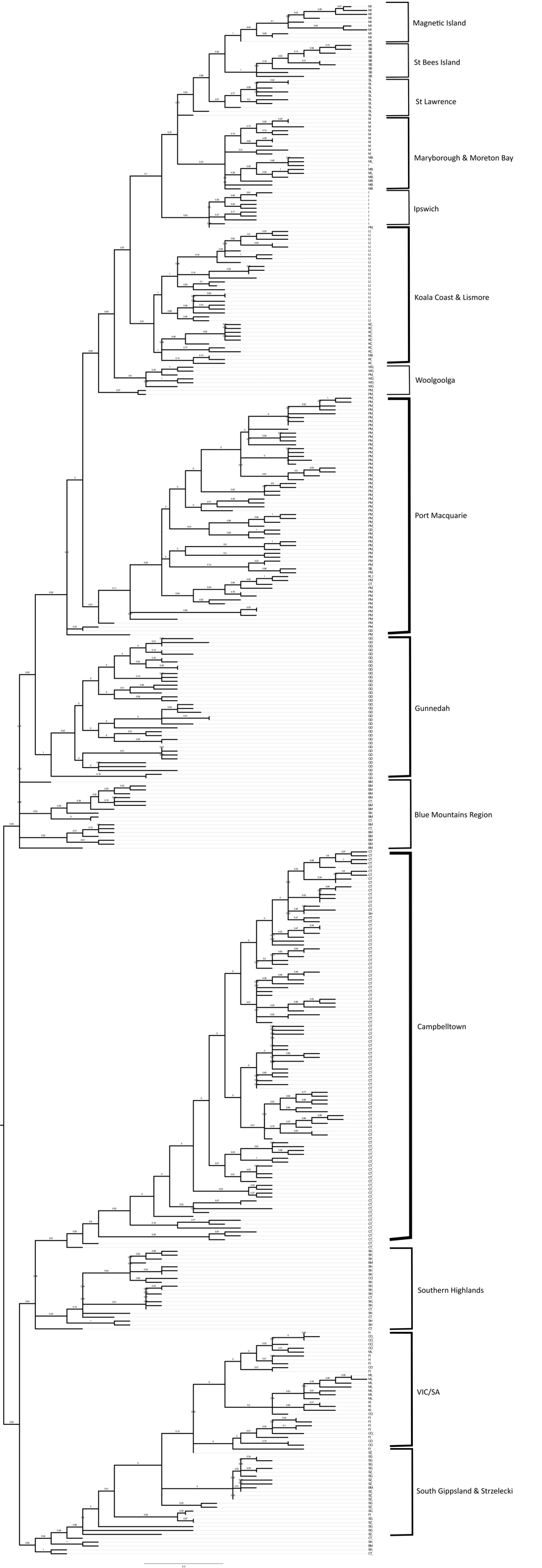

Supplement: Supplementary file 3 — Supplementary Figure 2. a) [file 41437_2018_144_MOESM3_ESM.pdf]

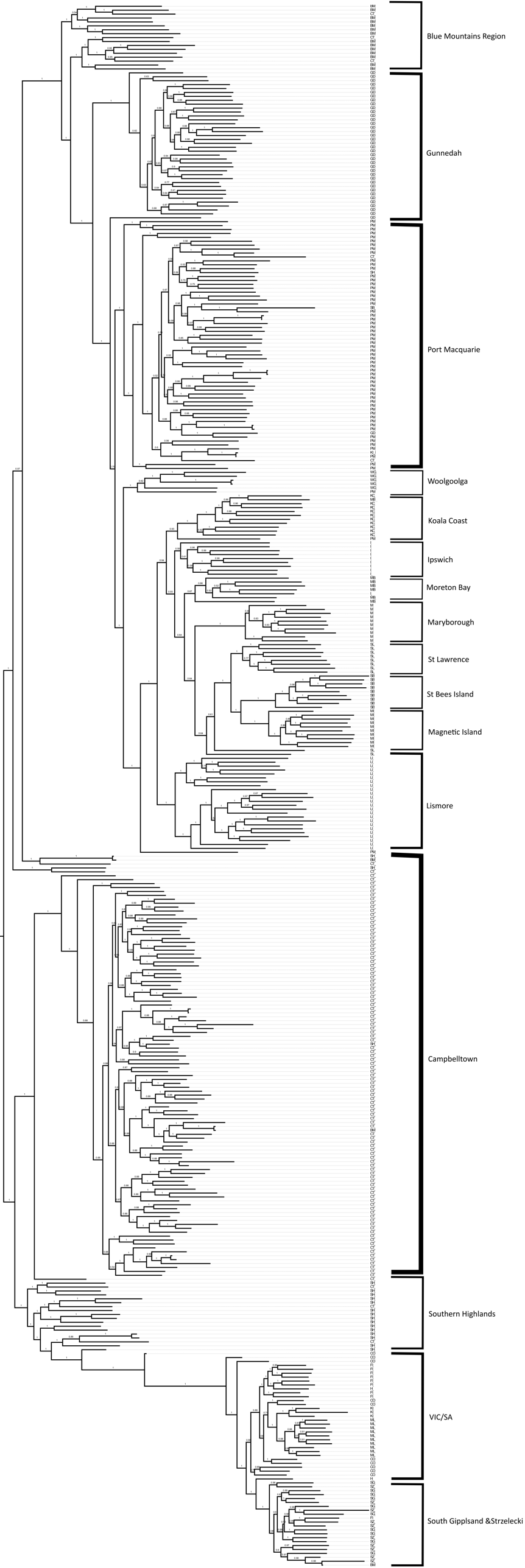

Supplement: Supplementary file 4 — Supplementary Figure 2. b) [file 41437_2018_144_MOESM4_ESM.pdf]

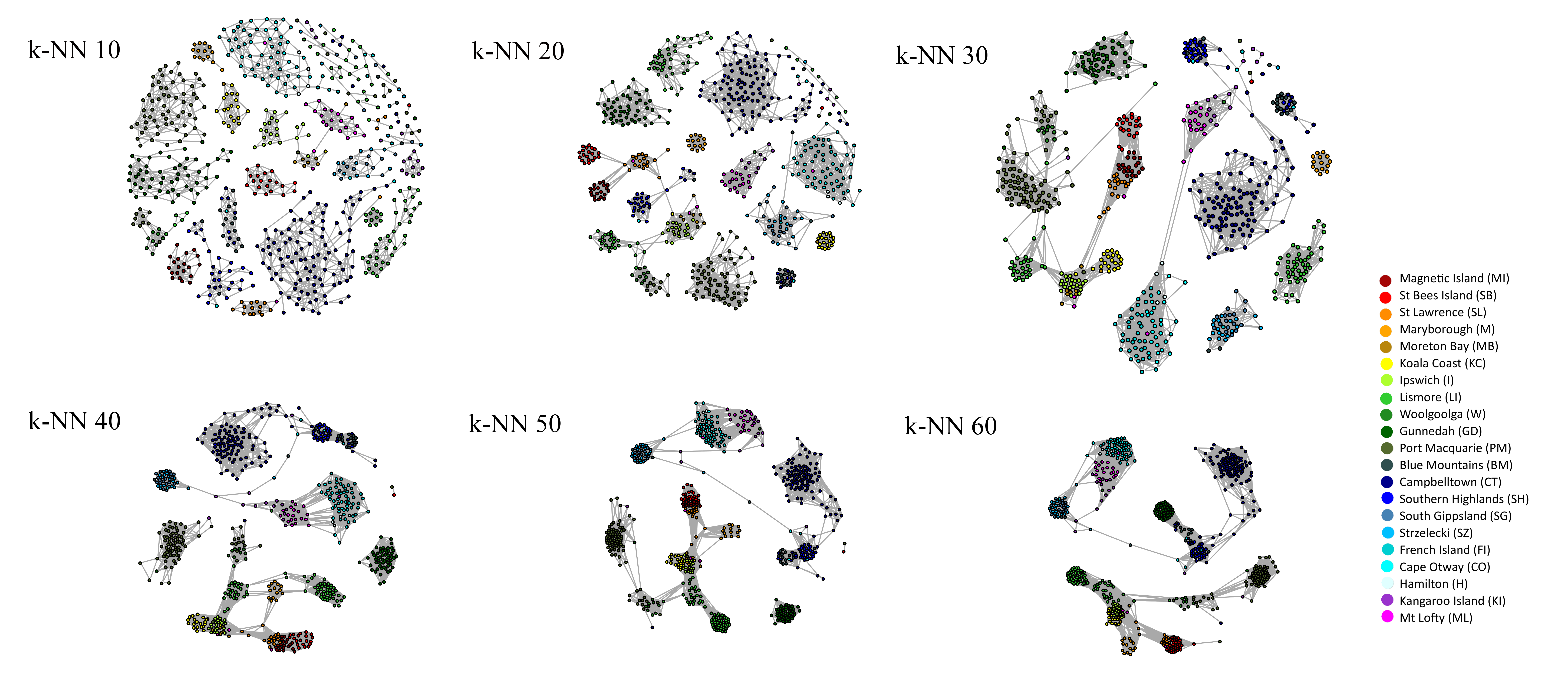

Supplement: Supplementary file 5 — Supplementary Figure 3 [file 41437_2018_144_MOESM5_ESM.png]

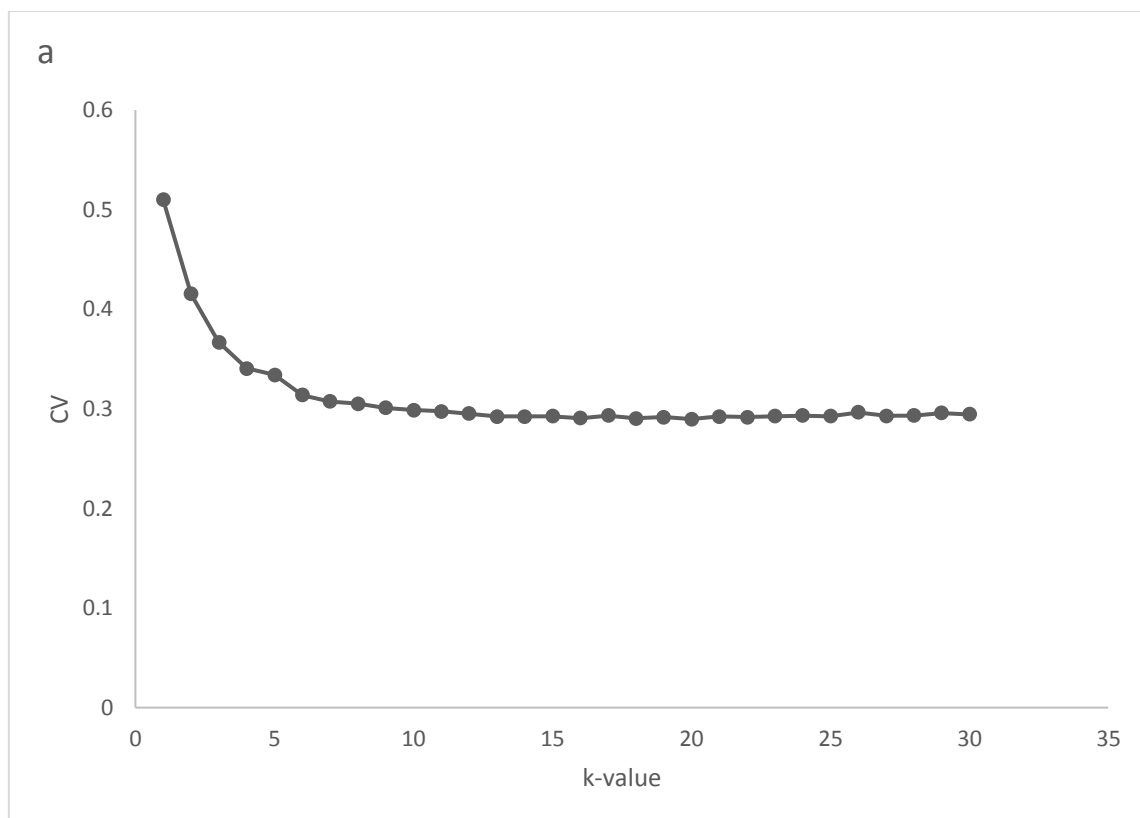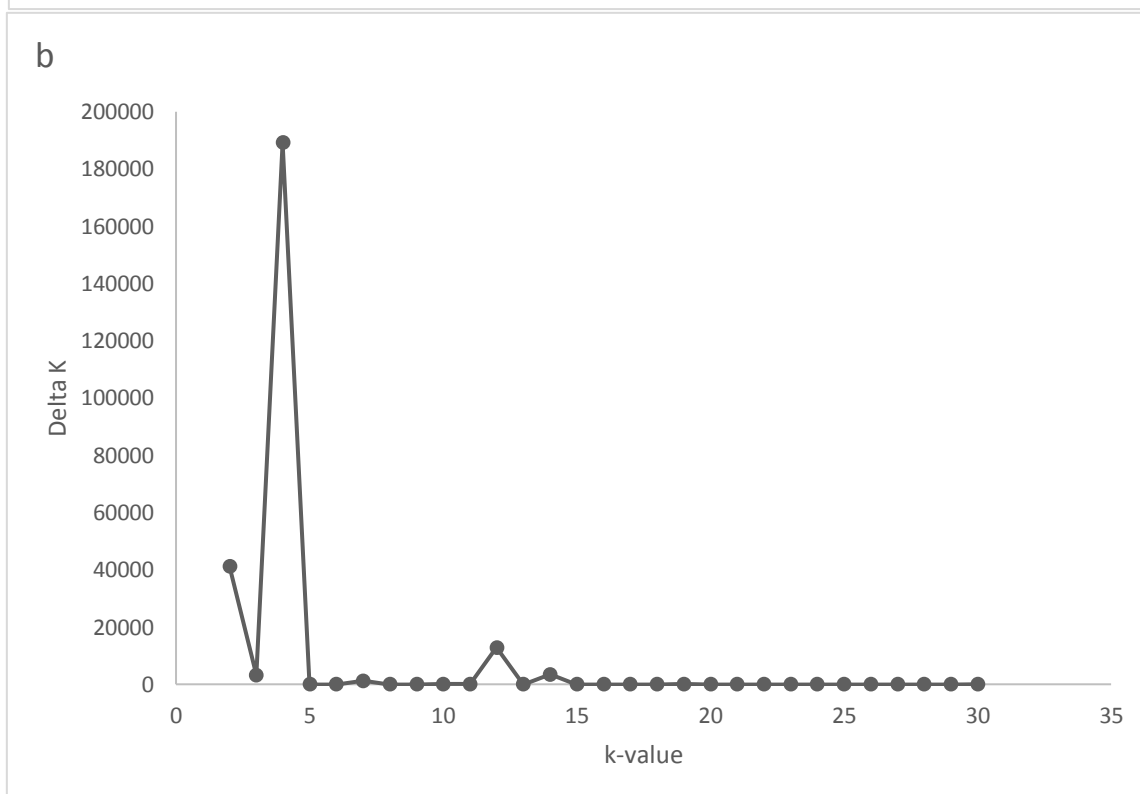

Supplement: Supplementary file 6 — Supplementary Figure 4 [file 41437_2018_144_MOESM6_ESM.pdf]
