# Supplementary material for: Genomic comparisons reveal biogeographic and anthropogenic impacts in the koala (Phascolarctos cinereus): a dietary-specialist species distributed across heterogeneous environments
Source: Heredity (Edinb). 2018 Sep 12;122(5):525–44. doi: 10.1038/s41437-018-0144-4 (PMC6461856; doi:10.1038/s41437-018-0144-4)
Supplement: Supplementary file 7 — Supplementary Table 1 [file 41437_2018_144_MOESM7_ESM.docx]

**Supplementary Table 1.** Hierarchical analysis of molecular variances between multiple groupings of populations based on: **a)** Northern clade and Southern clade, **b)** Four Groupings (1-[Magnetic Island, St Bees Island, St Lawrence, Maryborough, Moreton Bay, Koala Coast, Ipswich, Lismore], 2- [Woolgoolga, Port Macquarie], 3-[Gunnedah, Blue Mountains, Campbelltown, Southern Highlands], 4- [South Gippsland, Strzelecki, French Island, Cape Otway, Hamilton, Mt Lofty, Kangaroo Island]), **c)** Five Groups (based on mtDNA genetic divides proposed by Neaves *et al*.2016), **d)** Ten groups (1-[Magnetic Island, St Bees Island, St Lawrence, 2- [Maryborough], 3- [Moreton Bay, Koala Coast, Ipswich], 4- [Lismore], 5- [Woolgoolga, Port Macquarie], 6-[Gunnedah], 7- [Blue Mountains, Campbelltown, Southern Highlands], 8- [South Gippsland, Strzelecki], 9- [French Island, Cape Otway, Hamilton], 10- [Mt Lofty, Kangaroo Island])

1. Northern clade and Southern clade, based on Netview R clustering at k-NN60 ([1- Magnetic Island, St Bees Island, St Lawrence, Maryborough, Moreton Bay, Koala Coast, Ipswich, Lismore, Woolgoolga, Port Macquarie, Gunnedah], 2- [Blue Mountains, Campbelltown, Southern Highlands, South Gippsland, Strzelecki, French Island, Cape Otway, Hamilton, Mt Lofty, Kangaroo Island])

| **Source** | **df** | **SS** | **MS** | **Est. Var.** | **%** |
| --- | --- | --- | --- | --- | --- |
| **Among Regions** | 1 | 91558.227 | 91558.227 | 95.419 | 0.104935856 |
| **Among Pops** | 19 | 259017.297 | 13632.489 | 196.773 | 0.203872608 |
| **Among Indiv** | 705 | 692311.856 | 982.003 | 319.170 | 0.293940053 |
| **Within Indiv** | 726 | 249499.000 | 343.663 | 343.663 | 0.397251483 |
| **Total** | 1451 | 1292386.380 |  | 955.025 | 1 |

1. Four Groupings, based on a combination of clustering observed in Netview R clustering at k-NN50, and levels of Admixture observed in Figure 3 (1-[Magnetic Island, St Bees Island, St Lawrence, Maryborough, Moreton Bay, Koala Coast, Ipswich, Lismore], 2- [Woolgoolga, Port Macquarie], 3-[Gunnedah, Blue Mountains, Campbelltown, Southern Highlands], 4- [South Gippsland, Strzelecki, French Island, Cape Otway, Hamilton, Mt Lofty, Kangaroo Island])

| **Source** | **df** | **SS** | **MS** | **Est. Var.** | **%** |
| --- | --- | --- | --- | --- | --- |
| **Among Regions** | 3 | 159972.7747 | 53324.25825 | 114.0912 | 0.131451 |
| **Among Pops** | 17 | 135797.5209 | 7988.089465 | 140.6942 | 0.162102 |
| **Among Indiv** | 637 | 556675.1086 | 873.9012694 | 260.7515 | 0.300427 |
| **Within Indiv** | 658 | 231878 | 352.3981763 | 352.3982 | 0.406019 |
| **Total** | 1315 | 1084323.404 |  | 867.9351 | 1 |

1. Five Groups - based on mtDNA genetic divides proposed by Neaves et al 2016 (1-[ Magnetic Island, St Bees Island,], 2- [St Lawrence, Maryborough], 3- [Moreton Bay, Koala Coast, Ipswich, Lismore], 4- [Woolgoolga, Port Macquarie, Gunnedah], 5- [Blue Mountains, Campbelltown, Southern Highlands, South Gippsland, Strzelecki, French Island, Cape Otway, Hamilton, Mt Lofty, Kangaroo Island])

| **Source** | **df** | **SS** | **MS** | **Est. Var.** | **%** |
| --- | --- | --- | --- | --- | --- |
| **Among Regions** | 4 | 127251.2747 | 31812.81868 | 72.26063 | 0.08376 |
| **Among Pops** | 16 | 168519.0209 | 10532.43881 | 177.2983 | 0.205513 |
| **Among Indiv** | 637 | 556675.1086 | 873.9012694 | 260.7515 | 0.302248 |
| **Within Indiv** | 658 | 231878 | 352.3981763 | 352.3982 | 0.408479 |
| **Total** | 1315 | 1084323.404 |  | 862.7086 | 1 |

1. Ten groups – based approximately on Netview R clustering at k-NN20 (1-[Magnetic Island, St Bees Island, St Lawrence, 2- [Maryborough], 3- [Moreton Bay, Koala Coast, Ipswich], 4- [Lismore], 5- [Woolgoolga, Port Macquarie], 6-[Gunnedah], 7- [Blue Mountains, Campbelltown, Southern Highlands], 8- [South Gippsland, Strzelecki], 9- [French Island, Cape Otway, Hamilton], 10- [Mt Lofty, Kangaroo Island])

| **Source** | **df** | **SS** | **MS** | **Est. Var.** | **%** |
| --- | --- | --- | --- | --- | --- |
| **Among Regions** | 9 | 230247.575 | 25583.064 | 116.348 | 0.136738794 |
| **Among Pops** | 11 | 65522.721 | 5956.611 | 121.383 | 0.142655185 |
| **Among Indiv** | 637 | 556675.109 | 873.901 | 260.752 | 0.306449024 |
| **Within Indiv** | 658 | 231878.000 | 352.398 | 352.398 | 0.414156996 |
| **Total** | 1315 | 1084323.404 |  | 850.881 | 1 |
