# Supplementary material for: Genomic comparisons reveal biogeographic and anthropogenic impacts in the koala (Phascolarctos cinereus): a dietary-specialist species distributed across heterogeneous environments
Source: Heredity (Edinb). 2018 Sep 12;122(5):525–44. doi: 10.1038/s41437-018-0144-4 (PMC6461856; doi:10.1038/s41437-018-0144-4)
Supplement: Supplementary file 8 — Supplementary Table 2 [file 41437_2018_144_MOESM8_ESM.docx]

**Supplementary** **Table 2.** F_ST_ values between pair of populations with n>10, calculated using Meirman’s approach (2004) based on 999 permutations (bottom left matrix)

|  |  | **1** | **2** | **3** | **4** | **6** | **7** | **8** | **10** | **11** | **12** | **13** | **14** | **15** | **16** | **17** | **18** | **20** | **21** |
| --- | --- | --- | --- | --- | --- | --- | --- | --- | --- | --- | --- | --- | --- | --- | --- | --- | --- | --- | --- |
| **1** | **Magnetic Island** | 0 |  |  |  |  |  |  |  |  |  |  |  |  |  |  |  |  |  |
| **2** | **St Bees Island** | 0.131 | 0 |  |  |  |  |  |  |  |  |  |  |  |  |  |  |  |  |
| **3** | **St Lawrence** | 0.05 | 0.08 | 0 |  |  |  |  |  |  |  |  |  |  |  |  |  |  |  |
| **4** | **Maryborough** | 0.187 | 0.217 | 0.117 | 0 |  |  |  |  |  |  |  |  |  |  |  |  |  |  |
| **6** | **Koala Coast** | 0.16 | 0.19 | 0.091 | 0.113 | 0 |  |  |  |  |  |  |  |  |  |  |  |  |  |
| **7** | **Ipswich** | 0.125 | 0.152 | 0.05 | 0.078 | 0.022 | 0 |  |  |  |  |  |  |  |  |  |  |  |  |
| **8** | **Lismore** | 0.178 | 0.194 | 0.11 | 0.142 | 0.08 | 0.053 | 0 |  |  |  |  |  |  |  |  |  |  |  |
| **10** | **Gunnedah** | 0.245 | 0.258 | 0.18 | 0.211 | 0.177 | 0.15 | 0.19 | 0 |  |  |  |  |  |  |  |  |  |  |
| **11** | **Port Macquarie** | 0.22 | 0.224 | 0.149 | 0.197 | 0.148 | 0.113 | 0.146 | 0.133 | 0 |  |  |  |  |  |  |  |  |  |
| **12** | **Blue Mountains** | 0.224 | 0.246 | 0.154 | 0.187 | 0.139 | 0.111 | 0.153 | 0.081 | 0.105 | 0 |  |  |  |  |  |  |  |  |
| **13** | **Campbelltown** | 0.353 | 0.365 | 0.298 | 0.338 | 0.299 | 0.267 | 0.296 | 0.245 | 0.256 | 0.134 | 0 |  |  |  |  |  |  |  |
| **14** | **Southern Highlands** | 0.289 | 0.311 | 0.225 | 0.265 | 0.207 | 0.182 | 0.222 | 0.16 | 0.185 | 0.023 | 0.094 | 0 |  |  |  |  |  |  |
| **15** | **South Gippsland** | 0.383 | 0.419 | 0.328 | 0.353 | 0.29 | 0.272 | 0.333 | 0.285 | 0.322 | 0.108 | 0.283 | 0.145 | 0 |  |  |  |  |  |
| **16** | **Strzelecki** | 0.428 | 0.459 | 0.37 | 0.395 | 0.349 | 0.33 | 0.354 | 0.289 | 0.332 | 0.143 | 0.286 | 0.193 | -0.137 | 0 |  |  |  |  |
| **17** | **French Island** | 0.238 | 0.257 | 0.178 | 0.156 | 0.179 | 0.177 | 0.296 | 0.207 | 0.274 | 0.012 | 0.247 | 0.087 | -0.147 | -0.168 | 0 |  |  |  |
| **18** | **Cape Otway** | 0.351 | 0.373 | 0.295 | 0.312 | 0.285 | 0.267 | 0.327 | 0.266 | 0.307 | 0.1 | 0.265 | 0.139 | -0.112 | -0.096 | -0.144 | 0 |  |  |
| **20** | **Kangaroo Island** | 0.175 | 0.206 | 0.101 | 0.067 | 0.088 | 0.094 | 0.231 | 0.138 | 0.216 | -0.095 | 0.21 | 0.008 | -0.264 | -0.213 | -0.419 | -0.228 | 0 |  |
| **21** | **Mt Lofty** | 0.331 | 0.362 | 0.274 | 0.298 | 0.248 | 0.225 | 0.293 | 0.263 | 0.29 | 0.103 | 0.272 | 0.143 | -0.134 | -0.057 | -0.139 | -0.132 | -0.243 | 0 |
