# Supplementary material for: Genomic comparisons reveal biogeographic and anthropogenic impacts in the koala (Phascolarctos cinereus): a dietary-specialist species distributed across heterogeneous environments
Source: Heredity (Edinb). 2018 Sep 12;122(5):525–44. doi: 10.1038/s41437-018-0144-4 (PMC6461856; doi:10.1038/s41437-018-0144-4)
Supplement: Supplementary file 9 — Supplementary Table 3 [file 41437_2018_144_MOESM9_ESM.pdf]

Fst Outliers:	Magnetic Island	St Bees Island	St Lawrence	Maryborough	Moreton Bay	Koala Coast	Ipswich	Lismore	Woolgoolga	Port Macquarie	Gunnedah	Blue Mountains	Campbelltown
**Magnetic Island**													
**St Bees Island**	0.268												
**St Lawrence**	0.162	0.168											
**Maryborough**	0.364	0.405	0.241										
**Moreton Bay**	0.362	0.294	0.164	0.226									
**Koala Coast**	0.32	0.285	0.191	0.213	0.091								
**Ipswich**	0.275	0.24	0.136	0.201	0.052	0.082							
**Lismore**	0.355	0.339	0.248	0.252	0.129	0.154	0.134						
**Woolgoolga**	0.503	0.495	0.347	0.365	0.278	0.294	0.247	0.27					
**Port Macquarie**	0.565	0.554	0.481	0.486	0.443	0.444	0.405	0.39	0.19				
**Gunnedah**	0.468	0.439	0.375	0.412	0.357	0.339	0.292	0.305	0.136	0.223			
**Blue Mountains**	0.595	0.57	0.485	0.501	0.435	0.442	0.407	0.412	0.16	0.157	0.247		
**Campbelltown**	0.705	0.697	0.658	0.674	0.655	0.646	0.623	0.611	0.473	0.41	0.485	0.246	
**Southern Highlands**	0.698	0.685	0.617	0.637	0.595	0.588	0.554	0.548	0.351	0.323	0.412	0.126	0.13

Fst Neutral:	Magnetic Island	St Bees Island	St Lawrence	Maryborough	Moreton Bay	Koala Coast	Ipswich	Lismore	Woolgoolga	Port Macquarie	Gunnedah	Blue Mountains	Campbelltown
**Magnetic Island**													
**St Bees Island**	0.172												
**St Lawrence**	0.097	0.117											
**Maryborough**	0.235	0.262	0.152										
**Moreton Bay**	0.186	0.22	0.092	0.145									
**Koala Coast**	0.192	0.224	0.123	0.166	0.081								
**Ipswich**	0.156	0.185	0.086	0.136	0.057	0.06							
**Lismore**	0.188	0.208	0.125	0.164	0.098	0.102	0.076						
**Woolgoolga**	0.292	0.347	0.223	0.275	0.159	0.177	0.171	0.207					
**Port Macquarie**	0.27	0.288	0.205	0.24	0.173	0.208	0.191	0.195	0.222				
**Gunnedah**	0.199	0.206	0.137	0.186	0.123	0.143	0.114	0.133	0.186	0.146			
**Blue Mountains**	0.218	0.246	0.149	0.198	0.115	0.145	0.123	0.148	0.127	0.121	0.106		
**Campbelltown**	0.309	0.322	0.255	0.3	0.243	0.265	0.24	0.25	0.311	0.236	0.214	0.143	
**Southern Highlands**	0.258	0.284	0.199	0.254	0.183	0.186	0.163	0.195	0.189	0.197	0.165	0.061	0.127

Difference													
	**Magnetic Island**	**St Bees Island**	**St Lawrence**	**Maryborough**	**Moreton Bay**	**Koala Coast**	**Ipswich**	**Lismore**	**Woolgoolga**	**Port Macquarie**	**Gunnedah**	**Blue Mountains**	**Campbelltown**
**Magnetic Island**	0	0	0	0	0	0	0	0	0	0	0	0	0
**St Bees Island**	0.096	0	0	0	0	0	0	0	0	0	0	0	0
**St Lawrence**	0.065	0.051	0	0	0	0	0	0	0	0	0	0	0
**Maryborough**	0.129	0.143	0.089	0	0	0	0	0	0	0	0	0	0
**Moreton Bay**	0.176	0.074	0.072	0.081	0	0	0	0	0	0	0	0	0
**Koala Coast**	0.128	0.061	0.068	0.047	0.01	0	0	0	0	0	0	0	0
**Ipswich**	0.119	0.055	0.05	0.065	-0.005	0.022	0	0	0	0	0	0	0
**Lismore**	0.167	0.131	0.123	0.088	0.031	0.052	0.058	0	0	0	0	0	0
**Woolgoolga**	0.211	0.148	0.124	0.09	0.119	0.117	0.076	0.063	0	0	0	0	0
**Port Macquarie**	0.295	0.266	0.276	0.246	0.27	0.236	0.214	0.195	-0.032	0	0	0	0
**Gunnedah**	0.269	0.233	0.238	0.226	0.234	0.196	0.178	0.172	-0.05	0.077	0	0	0
**Blue Mountains**	0.377	0.324	0.336	0.303	0.32	0.297	0.284	0.264	0.033	0.036	0.141	0	0
**Campbelltown**	0.396	0.375	0.403	0.374	0.412	0.381	0.383	0.361	0.162	0.174	0.271	0.103	0
**Southern Highlands**	0.44	0.401	0.418	0.383	0.412	0.402	0.391	0.353	0.162	0.126	0.247	0.065	0.003

Combining Bioregions 3 and 4 together.

Bio Regions	1	2	3	4	
1	0.07				
2	0.11	0.05			
3	0.23	0.18	0.00		
4	0.39	0.35	0.15	0.06	

Bio Regions	1	2	3	4	5
1	0.07				
2	0.11	0.05			
3	0.22	0.16	0.00		
4	0.24	0.21	0.01	-	
5	0.39	0.35	0.11	0.22	0.06
					SD
	Within Bioregion =			0.04	0.030641747
	Between bioregion =			0.2	0.113019172
