# Supplementary material for: Genomic comparisons reveal biogeographic and anthropogenic impacts in the koala (Phascolarctos cinereus): a dietary-specialist species distributed across heterogeneous environments
Source: Heredity (Edinb). 2018 Sep 12;122(5):525–44. doi: 10.1038/s41437-018-0144-4 (PMC6461856; doi:10.1038/s41437-018-0144-4)
Supplement: Supplementary file 10 — Supplementary Table 4 [file 41437_2018_144_MOESM10_ESM.pdf]

NAME	% IDENTICAL SITES	% PAIRWISE IDENTITY	BIT-SCORE	DESCRIPTION	E VALUE	GRADE	HIT END	HIT START	MEAN COVERAGE	MOLECULE TYPE	ORGANISM	QUERY	QUERY COVERAGE	QUERY END	QUERY START
**XM_020982353**	100.00%	100.00%	2128.46	PREDICTED: Phascolarctos cinereus PARP1 binding protein (PARPBP), transcript variant X1, mRNA	0	64.20%	2625	1474	2	mRNA	PREDICTED: Phascolarctos cinereus	MSTS01000014	28.46%	2433	1282
**XM_020985131**	100.00%	100.00%	5644.48	PREDICTED: Phascolarctos cinereus zinc finger protein 423 (ZNF423), transcript variant X4, mRNA	0	87.60%	3553	498	2	mRNA	PREDICTED: Phascolarctos cinereus	MSTS01000020	75.10%	4069	1014
**XR_002324717**	100.00%	100.00%	281.811	PREDICTED: Phascolarctos cinereus uncharacterized LOC110209041 (LOC110209041), ncRNA	9.10E-71	51.90%	233	82	2	RNA	PREDICTED: Phascolarctos cinereus	MSTS01000029	3.74%	3416	3265
**XM_020987784**	100.00%	100.00%	331.671	PREDICTED: Phascolarctos cinereus zinc finger CCHC-type containing 11 (ZCCHC11), transcript variant X6, mRNA	8.91E-86	52.20%	1368	1190	2	mRNA	PREDICTED: Phascolarctos cinereus	MSTS01000030	4.40%	2832	2654
**XM_020988738**	100.00%	100.00%	274.424	PREDICTED: Phascolarctos cinereus dimethylarginine dimethylaminohydrolase 1 (DDAH1), mRNA	1.52E-68	51.80%	925	778	2	mRNA	PREDICTED: Phascolarctos cinereus	MSTS01000033	3.66%	3747	3600
**XM_020996570**	100.00%	100.00%	300.278	PREDICTED: Phascolarctos cinereus HscB mitochondrial iron-sulfur cluster cochaperone (HSCB), mRNA	2.50E-76	52.00%	845	684	2	mRNA	PREDICTED: Phascolarctos cinereus	MSTS01000065	4.00%	1329	1168
**XM_020996978**	100.00%	100.00%	7470.82	PREDICTED: Phascolarctos cinereus ligand dependent nuclear receptor corepressor (LCOR), transcript variant X6, mRNA	0	100.00%	5532	1488	2	mRNA	PREDICTED: Phascolarctos cinereus	MSTS01000066	100.00%	4045	1
**XM_020997531**	100.00%	100.00%	206.098	PREDICTED: Phascolarctos cinereus Rho GTPase activating protein 27 (ARHGAP27), transcript variant X4, mRNA	5.64E-48	51.40%	1489	1379	2	mRNA	PREDICTED: Phascolarctos cinereus	MSTS01000068	2.73%	2148	2038
**XM_021005187**	100.00%	100.00%	1519.07	PREDICTED: Phascolarctos cinereus DND microRNA-mediated repression inhibitor 1 (DND1), transcript variant X2, mRNA	0	60.20%	1752	931	2	mRNA	PREDICTED: Phascolarctos cinereus	MSTS01000108	20.33%	2609	1788
**XM_021005774**	100.00%	100.00%	346.444	PREDICTED: Phascolarctos cinereus mitogen-activated protein kinase kinase kinase 13 (MAP3K13), mRNA	3.18E-90	52.30%	1798	1612	2	mRNA	PREDICTED: Phascolarctos cinereus	MSTS01000110	4.60%	3962	3776
**XM_021007975**	100.00%	100.00%	1190.36	PREDICTED: Phascolarctos cinereus iron-sulfur cluster assembly enzyme (ISCU), mRNA	0	57.90%	1087	444	2	mRNA	PREDICTED: Phascolarctos cinereus	MSTS01000127	15.83%	3813	3170
**XM_020963593**	100.00%	100.00%	1404.57	PREDICTED: Phascolarctos cinereus Vac14, PIKFYVE complex component (VAC14), transcript variant X2, mRNA	0	59.30%	3245	2486	2	mRNA	PREDICTED: Phascolarctos cinereus	MSTS01000147	18.68%	2516	1757

**XM_020965168**	100.00%	100.00%	3171.82	PREDICTED: Phascolarctos cinereus SH3 domain containing ring finger 3 (SH3RF3), transcript variant X3, mRNA	0	71.10%	7284	5568	2	mRNA	PREDICTED: Phascolarctos cinereus	MSTS01000159	42.20%	4069	2353
**XM_020970155**	100.00%	100.00%	1801.6	PREDICTED: Phascolarctos cinereus SET domain containing 1B (SETD1B), mRNA	0	62.00%	5385	4411	2	mRNA	PREDICTED: Phascolarctos cinereus	MSTS01000203	23.96%	4069	3095
**XM_020970722**	100.00%	100.00%	614.208	PREDICTED: Phascolarctos cinereus MAS related GPR family member F (MRGPRF), transcript variant X1, mRNA	7.90E-171	54.10%	332	1	2	mRNA	PREDICTED: Phascolarctos cinereus	MSTS01000210	8.16%	3734	3403
**XM_020971757**	100.00%	100.00%	575.428	PREDICTED: Phascolarctos cinereus RNA binding motif protein, X-linked (RBMX), transcript variant X4, mRNA	3.73E-159	53.80%	311	1	2	mRNA	PREDICTED: Phascolarctos cinereus	MSTS01000220	7.64%	2593	2283
**XM_020973703**	100.00%	100.00%	1655.72	PREDICTED: Phascolarctos cinereus family with sequence similarity 208 member B (FAM208B), transcript variant X3, mRNA	0	61.10%	2783	1888	2	mRNA	PREDICTED: Phascolarctos cinereus	MSTS01000246	22.11%	1082	187
**XM_020976478**	100.00%	100.00%	246.725	PREDICTED: Phascolarctos cinereus kinesin family member 4A (KIF4A), transcript variant X2, mRNA	3.32E-60	51.60%	1339	1207	2	mRNA	PREDICTED: Phascolarctos cinereus	MSTS01000295	3.27%	384	252
**XM_020978137**	100.00%	100.00%	267.038	PREDICTED: Phascolarctos cinereus mitogen-activated protein kinase kinase 7 (MAP2K7), transcript variant X3, mRNA	2.54E-66	51.80%	314	171	2	mRNA	PREDICTED: Phascolarctos cinereus	MSTS01000331	3.54%	364	221
**XR_002322692**	100.00%	100.00%	281.811	PREDICTED: Phascolarctos cinereus uncharacterized LOC110202296 (LOC110202296), ncRNA	9.10E-71	51.90%	302	151	2	RNA	PREDICTED: Phascolarctos cinereus	MSTS01000340	3.74%	450	299
**XM_020981704**	99.80%	99.80%	1134.96	PREDICTED: Phascolarctos cinereus ectonucleoside triphosphate diphosphohydrolase 8-like (LOC110205253), mRNA	0	57.60%	618	1	2	mRNA	PREDICTED: Phascolarctos cinereus	MSTS01000012	15.27%	883	267
**AC156035**	98.80%	98.80%	147.006	Mus musculus BAC clone RP23-302M20 from chromosome 12, complete sequence	3.46E-30	50.40%	87760	87679	2	DNA	Mus musculus	MSTS01000195	2.02%	4069	3988
**AL355599**	98.20%	98.20%	98.9927	Human DNA sequence from clone RP11-476C5 on chromosome 13, complete sequence	9.79E-16	24.80%	57377	57322	2	DNA	Human DNA	MSTS01000192	1.41%	377	321
**LL914865**	97.80%	97.80%	159.932	Schistocephalus solidus genome assembly S_solidus_NST_G2 ,scaffold SSLN_scaffold0013536	4.45E-34	50.10%	8032	7940	2	DNA	Schistocephalus solidus	MSTS01000070	2.26%	2707	2616
**AC150277**	97.10%	97.10%	292.891	Mus musculus BAC clone RP23-97E12 from 18, complete sequence	4.21E-74	50.70%	144483	144311	2	DNA	Mus musculus	MSTS01000050	4.28%	387	214

**XM_020990166**	96.80%	96.80%	2630.75	PREDICTED: Phascolarctos cinereus ubiquitin specific peptidase 15 (USP15), transcript variant X2, mRNA	0	67.80%	2962	1379	2	mRNA	PREDICTED: Phascolarctos cinereus	MSTS01000005	38.74%	3326	1756
**KT496552**	95.90%	95.90%	1836.69	Aphelocoma californica x Aphelocoma woodhouseii voucher MLZ:59834 ultra conserved element locus uce-457 genomic sequence	0	61.90%	1477	345	2	DNA	Aphelocoma californica	MSTS01000123	27.82%	3056	1925
**BX928757**	94.60%	94.60%	276.271	Zebrafish DNA sequence from clone CH211-74M19 in linkage group 1, complete sequence	4.23E-69	49.40%	114276	114094	2	DNA	Zebrafish DNA	MSTS01000343	4.33%	458	283
**AC147769**	94.50%	94.50%	473.863	Homo sapiens chromosome 16 clone PCR-16A01, complete sequence	1.40E-128	51.00%	5365	5059	2	DNA	Homo sapiens	MSTS01000020	7.57%	566	259
**AC270641**	94.20%	94.20%	156.239	Pongo abelii chromosome 10 clone CH276-217H21, complete sequence	5.71E-33	48.40%	79679	79577	2	DNA	Pongo abelii	MSTS01000049	2.53%	4035	3934
**FN661652**	93.90%	93.90%	150.699	Dromiciops gliroides partial cyb5R2 gene	2.68E-31	48.20%	663	565	2	DNA	Dromiciops gliroides	MSTS01000207	2.43%	1208	1110
**AC129583**	93.50%	93.50%	339.057	Mus musculus chromosome 15, clone RP23-197P10, complete sequence	5.33E-88	49.60%	144961	144734	2	DNA	Mus musculus	MSTS01000103	5.60%	1049	822
**LK064674**	93.40%	93.40%	422.156	Apteryx australis mantelli genome assembly AptMant0, scaffold scaffold33	5.14E-113	50.20%	15074963	15074678	2	DNA	Apteryx australis	MSTS01000001	7.00%	889	605
**XM_020976889**	93.20%	93.20%	193.172	PREDICTED: Phascolarctos cinereus solute carrier family 22 member 7 (SLC22A7), transcript variant X2, mRNA	4.39E-44	48.20%	2583	2454	2	mRNA	PREDICTED: Phascolarctos cinereus	MSTS01000029	3.24%	1660	1529
**AC115508**	93.10%	93.10%	126.692	Rattus norvegicus 2 BAC CH230-355B17 (Children's Hospital Oakland Research Institute) complete sequence	4.51E-24	47.60%	153736	153651	2	DNA	Rattus norvegicus	MSTS01000089	2.11%	1427	1342
**XM_020976889**	93.10%	93.10%	191.325	PREDICTED: Phascolarctos cinereus solute carrier family 22 member 7 (SLC22A7), transcript variant X2, mRNA	1.58E-43	48.10%	2583	2454	2	mRNA	PREDICTED: Phascolarctos cinereus	MSTS01000185	3.19%	3408	3279
**XM_020976889**	93.00%	93.00%	187.632	PREDICTED: Phascolarctos cinereus solute carrier family 22 member 7 (SLC22A7), transcript variant X2, mRNA	2.04E-42	48.10%	2582	2454	2	mRNA	PREDICTED: Phascolarctos cinereus	MSTS01000080	3.15%	1332	1205
**FN661609**	92.90%	92.90%	183.939	Dromiciops gliroides partial ppef2 gene	2.64E-41	48.00%	210	85	2	DNA	Dromiciops gliroides	MSTS01000146	3.12%	1174	1048
**AB161647**	92.60%	92.60%	172.859	Cryptomeria japonica DNA, microsatellite locus, clone: CJS0584	5.69E-38	47.80%	434	313	2	DNA	Cryptomeria japonica	MSTS01000071	2.91%	890	773
**FN661609**	92.20%	92.20%	178.399	Dromiciops gliroides partial ppef2 gene	1.23E-39	47.60%	213	86	2	DNA	Dromiciops gliroides	MSTS01000166	3.07%	2663	2539
**CR352262**	91.80%	91.80%	119.306	Zebrafish DNA sequence from clone DKEY-39F23 in linkage group 24, complete sequence	7.55E-22	45.00%	215498	215414	2	DNA	Zebrafish DNA	MSTS01000050	2.09%	1022	938

**AC197856**	91.50%	91.50%	124.846	Gallus gallus BAC clone CH261-23M13 from chromosome z, complete sequence	1.62E-23	46.80%	89218	89125	2	DNA	Gallus gallus	MSTS01000154	2.19%	1654	1566
**XR_002321570**	90.90%	90.90%	638.214	PREDICTED: Phascolarctos cinereus uncharacterized LOC110197798 (LOC110197798), ncRNA	4.69E-178	51.30%	4519	4042	2	RNA	PREDICTED: Phascolarctos cinereus	MSTS01000174	11.72%	4069	3593
**KR270823**	89.60%	89.60%	213.485	Pinellia ternata chloroplast, complete genome	3.37E-50	46.90%	116674	116505	2	DNA	Pinellia ternata	MSTS01000146	4.13%	2314	2147
**XM_020969825**	89.60%	89.60%	86.0662	PREDICTED: Phascolarctos cinereus motile sperm domain containing 1 (MOSPD1), transcript variant X5, mRNA	7.65E-12	20.60%	2344	2278	2	mRNA	PREDICTED: Phascolarctos cinereus	MSTS01000154	1.65%	433	367
**CU463024**	89.40%	89.40%	202.405	Wallaby DNA sequence from clone MEKBa-201O22, complete sequence	7.29E-47	46.70%	37822	37664	2	DNA	Wallaby DNA	MSTS01000410	3.96%	3520	3360
**CU302407**	89.10%	89.10%	202.405	Wallaby DNA sequence from clone MEKBa-192E16, complete sequence	7.26E-47	46.60%	135934	135772	2	DNA	Wallaby DNA	MSTS01000076	4.03%	2844	2682
**XM_020963823**	89.00%	89.00%	122.999	PREDICTED: Phascolarctos cinereus potassium voltage-gated channel subfamily J member 5 (KCNJ5), transcript variant X2, mRNA	5.84E-23	45.60%	7256	7157	2	mRNA	PREDICTED: Phascolarctos cinereus	MSTS01000276	2.41%	3639	3542
**XM_020989059**	88.90%	88.90%	89.7595	PREDICTED: Phascolarctos cinereus galactose-3-O-sulfotransferase 2 (GAL3ST2), mRNA	5.92E-13	20.30%	2415	2344	2	mRNA	PREDICTED: Phascolarctos cinereus	MSTS01000385	1.77%	2156	2085
**CU062506**	88.80%	88.80%	291.044	Wallaby DNA sequence from clone MEKBa-183I7, complete sequence	1.50E-73	47.30%	150713	150475	2	DNA	Wallaby DNA	MSTS01000280	5.90%	2754	2517
**XM_007478826**	88.40%	88.40%	1062.94	PREDICTED: Monodelphis domestica polycomb group ring finger 5 (PCGF5), transcript variant X9, mRNA	0	55.20%	3849	2965	2	mRNA	PREDICTED: Monodelphis domestica	MSTS01000096	22.02%	4069	3174
**CR933563**	88.30%	88.30%	255.958	Wallaby DNA sequence from clone MEKBa-325O12, complete sequence	5.50E-63	46.80%	17230	17018	2	DNA	Wallaby DNA	MSTS01000224	5.25%	3767	3555
**FQ482147**	88.10%	88.10%	176.552	S.harrisii DNA sequence from clone bSHS-50J3, complete sequence	4.42E-39	45.90%	138936	138786	2	DNA	S.harrisii DNA	MSTS01000001	3.64%	1067	920
**AC154870**	88.10%	88.10%	427.696	Monodelphis domestica, clone XX-218A9, complete sequence	1.10E-114	48.60%	76114	75755	2	DNA	Monodelphis domestica,	MSTS01000160	9.03%	3893	3528
**XR_002328143**	87.70%	87.70%	73.1396	PREDICTED: Phascolarctos cinereus uncharacterized LOC110221502 (LOC110221502), ncRNA	5.96E-08	19.60%	175	111	2	RNA	PREDICTED: Phascolarctos cinereus	MSTS01000303	1.52%	3007	2946
**JN757271**	87.60%	87.60%	231.952	Cyprinus carpio clone 646048 microsatellite sequence	9.30E-56	46.30%	1098	891	2	DNA	Cyprinus carpio	MSTS01000014	4.89%	2790	2592

**CR936538**	87.60%	87.60%	468.323	Wallaby DNA sequence from clone MEKBa-43B7, complete sequence	6.50E-127	48.80%	53297	52894	2	DNA	Wallaby DNA	MSTS01000078	9.89%	3339	2938
**CU326409**	87.60%	87.60%	239.338	Wallaby DNA sequence from clone MEKBa-509L14, complete sequence	5.56E-58	46.30%	30974	30766	2	DNA	Wallaby DNA	MSTS01000134	5.06%	1026	821
**AF003528**	87.20%	87.20%	326.131	Homo sapiens X-linked anhidroitic ectodermal dysplasia protein gene (EDA), exon 2 and flanking repeat regions	4.15E-84	47.10%	10403	10117	2	DNA	Homo sapiens	MSTS01000295	7.08%	288	1
**CU326409**	87.10%	87.10%	233.798	Wallaby DNA sequence from clone MEKBa-509L14, complete sequence	2.58E-56	46.10%	30965	30757	2	DNA	Wallaby DNA	MSTS01000062	5.04%	3678	3474
**AM910993**	87.00%	87.00%	86.0662	Plasmodium knowlesi strain H chromosome 11, complete genome	7.66E-12	19.50%	1531905	1531831	2	DNA	Plasmodium knowlesi	MSTS01000381	1.89%	2496	2420
**CR933563**	86.90%	86.90%	243.031	Wallaby DNA sequence from clone MEKBa-325O12, complete sequence	4.30E-59	46.10%	157760	157539	2	DNA	Wallaby DNA	MSTS01000145	5.28%	1452	1238
**CR956372**	86.80%	86.80%	219.025	Wallaby DNA sequence from clone GRWB-15A6, complete sequence	7.24E-52	45.80%	52013	51817	2	DNA	Wallaby DNA	MSTS01000002	4.82%	1920	1725
**XM_012788973**	86.70%	86.70%	182.092	PREDICTED: Microcebus murinus zinc finger protein 423 (ZNF423), transcript variant X5, mRNA	9.42E-41	45.40%	525	362	2	mRNA	PREDICTED: Microcebus murinus	MSTS01000020	4.09%	1855	1691
**CU468218**	86.40%	86.40%	193.172	Wallaby DNA sequence from clone MEKBa-34H17, complete sequence	4.38E-44	45.30%	151576	151401	2	DNA	Wallaby DNA	MSTS01000299	4.33%	2605	2430
**LK064691**	85.50%	85.50%	483.096	Apteryx australis mantelli genome assembly AptMant0, scaffold scaffold105	2.32E-131	48.50%	1672595	1672129	2	DNA	Apteryx australis	MSTS01000011	11.53%	2365	1897
**CP020785**	85.50%	85.50%	281.811	Oryzias latipes strain HNI chromosome 7	9.10E-71	46.10%	870606	870334	2	DNA	Oryzias latipes	MSTS01000174	6.66%	3118	2848
**CR385056**	85.30%	85.30%	230.105	Wallaby DNA sequence from clone GRWB-43E9, complete sequence	3.34E-55	45.40%	37041	36816	2	DNA	Wallaby DNA	MSTS01000359	5.58%	455	229
**AP015035**	85.20%	85.20%	178.399	Vigna angularis var. angularis DNA, chromosome 2, almost complete sequence, cultivar: Shumari	1.23E-39	44.80%	44240800	44240624	2	DNA	Vigna angularis	MSTS01000056	4.37%	2474	2297
**CU326409**	85.00%	85.00%	252.265	Wallaby DNA sequence from clone MEKBa-509L14, complete sequence	7.09E-62	45.60%	31005	30754	2	DNA	Wallaby DNA	MSTS01000051	6.16%	2744	2496
**XM_020992468**	85.00%	85.00%	2213.41	PREDICTED: Phascolarctos cinereus DEAH-box helicase 15 (DHX15), mRNA	0	69.50%	2818	575	2	mRNA	PREDICTED: Phascolarctos cinereus	MSTS01000174	53.90%	3994	1802
**AC145250**	84.90%	84.90%	196.865	Notamacropus eugenii clone ME_KBa-232I1, complete sequence	3.39E-45	44.90%	103624	103429	2	DNA	Notamacropus eugenii	MSTS01000025	4.82%	2418	2223
**FP236732**	84.60%	84.60%	281.811	Wallaby DNA sequence from clone MEKBa-5M36, complete sequence	9.10E-71	45.90%	20564	20280	2	DNA	Wallaby DNA	MSTS01000361	7.10%	315	27

**BX957239**	84.50%	84.50%	147.006	Zebrafish DNA sequence from clone DKEY-173N20 in linkage group 19, complete sequence	3.46E-30	44.10%	28276	28125	2	DNA	Zebrafish DNA	MSTS01000397	3.69%	1090	941
**XM_020978573**	84.40%	84.40%	73.1396	PREDICTED: Phascolarctos cinereus HEPACAM family member 2 (HEPACAM2), mRNA	5.96E-08	18.10%	3792	3717	2	mRNA	PREDICTED: Phascolarctos cinereus	MSTS01000050	1.84%	990	916
**FP236744**	84.30%	84.30%	1127.58	Wallaby DNA sequence from clone MEKBa-575K20, complete sequence	0	56.60%	55521	54355	2	DNA	Wallaby DNA	MSTS01000038	28.90%	1184	9
**CT027995**	84.00%	84.00%	1267.92	Wallaby DNA sequence from clone MEKBa-583L10, complete sequence	0	58.30%	19154	17823	2	DNA	Wallaby DNA	MSTS01000062	32.61%	1343	19
**CT027995**	83.90%	83.90%	658.528	Wallaby DNA sequence from clone MEKBa-583L10, complete sequence	0	50.50%	18828	18133	2	DNA	Wallaby DNA	MSTS01000055	17.08%	698	4
**CU464025**	83.90%	83.90%	3214.29	Wallaby DNA sequence from clone MEKBa-210A8, complete sequence	0	84.50%	143539	140106	2	DNA	Wallaby DNA	MSTS01000255	85.08%	3496	42
**CR956372**	83.60%	83.60%	204.252	Wallaby DNA sequence from clone GRWB-15A6, complete sequence	2.03E-47	44.50%	82000	81783	2	DNA	Wallaby DNA	MSTS01000065	5.36%	1793	1576
**AY659987**	83.10%	83.10%	180.245	Macropus eugenii prion protein PrP (PRNP) gene, complete cds	3.42E-40	44.00%	40456	40247	2	DNA	Macropus eugenii	MSTS01000002	4.94%	3041	2841
**CR956354**	82.90%	82.90%	213.485	Wallaby DNA sequence from clone GRWB-143H14, complete sequence	3.37E-50	44.40%	3254	3015	2	DNA	Wallaby DNA	MSTS01000018	5.82%	1360	1124
**AY737497**	82.80%	82.80%	577.275	Macropus eugenii green-sensitive visual pigment (OPN1MW), CXorf2 (CXorf2), and TKT2-like protein (TKT2) genes, complete cds	1.04E-159	49.60%	54355	53699	2	DNA	Macropus eugenii	MSTS01000167	16.33%	2548	1885
**XR_002325761**	82.50%	82.50%	322.437	PREDICTED: Phascolarctos cinereus uncharacterized LOC110212811 (LOC110212811), transcript variant X2, ncRNA	5.36E-83	45.80%	1009	635	2	RNA	PREDICTED: Phascolarctos cinereus	MSTS01000001	9.07%	3384	3016
**CU012066**	82.50%	82.50%	1437.81	Wallaby DNA sequence from clone MEKBa-107A11, complete sequence	0	62.10%	41671	39995	2	DNA	Wallaby DNA	MSTS01000201	41.64%	1686	1
**AP015034**	82.40%	82.40%	383.377	Vigna angularis var. angularis DNA, chromosome 1, almost complete sequence, cultivar: Shumari	2.42E-101	46.70%	49226420	49225964	2	DNA	Vigna angularis	MSTS01000112	10.91%	3400	2958
**CU012066**	82.40%	82.40%	1844.08	Wallaby DNA sequence from clone MEKBa-107A11, complete sequence	0	68.30%	42142	39967	2	DNA	Wallaby DNA	MSTS01000252	54.26%	4049	1853
**CR925799**	81.90%	81.90%	272.578	Wallaby DNA sequence from clone GRWB-66O5, complete sequence	5.47E-68	45.10%	37931	37595	2	DNA	Wallaby DNA	MSTS01000349	8.27%	647	312
**CR933563**	81.70%	81.70%	315.051	Wallaby DNA sequence from clone MEKBa-325O12, complete sequence	8.97E-81	45.60%	30605	30219	2	DNA	Wallaby DNA	MSTS01000032	9.62%	2328	1938

**CP020785**	81.70%	81.70%	239.338	Oryzias latipes strain HNI chromosome 7	5.52E-58	44.50%	11027500	11027212	2	DNA	Oryzias latipes	MSTS01000120	7.28%	4028	3735
**JN251945**	81.60%	81.60%	89.7595	Macropus eugenii clone MEB1-9B19 uterine-secreted microprotein 1 (USM1) and uterine-secreted microprotein 2 (USM2) genes, complete cds; and elongation protein 3-like protein (ELP3) gene, partial cds	5.89E-13	17.20%	7982	7870	2	DNA	Macropus eugenii	MSTS01000049	2.72%	3817	3708
**LN595317**	81.50%	81.50%	586.508	Cyprinus carpio genome assembly common carp genome ,scaffold 000006154	1.72E-162	49.70%	4230	3496	2	DNA	Cyprinus carpio	MSTS01000032	18.01%	3702	2970
**FP104545**	81.40%	81.40%	298.431	Wallaby DNA sequence from clone MEKBa-293I1, complete sequence	9.04E-76	45.30%	105610	105230	2	DNA	Wallaby DNA	MSTS01000004	9.17%	2280	1908
**CU012066**	81.40%	81.40%	2191.25	Wallaby DNA sequence from clone MEKBa-107A11, complete sequence	0	74.50%	42178	39421	2	DNA	Wallaby DNA	MSTS01000168	67.63%	2931	191
**AL669896**	81.30%	81.30%	278.118	Mouse DNA sequence from clone RP23-477F4 on chromosome 2, complete sequence	1.18E-69	45.00%	102719	102363	2	DNA	Mouse DNA	MSTS01000032	8.65%	2447	2096
**FP104544**	81.20%	81.20%	252.265	Wallaby DNA sequence from clone MEKBa-163H18, complete sequence	7.12E-62	44.50%	68922	68600	2	DNA	Wallaby DNA	MSTS01000071	7.93%	537	216
**CU463152**	81.20%	81.20%	303.971	Wallaby DNA sequence from clone MEKBa-285B7, complete sequence	1.94E-77	45.30%	58181	57797	2	DNA	Wallaby DNA	MSTS01000220	9.41%	4053	3672
**CT573039**	80.80%	80.80%	475.709	Wallaby DNA sequence from clone MEKBa-403O8, complete sequence	3.89E-129	48.10%	8159	7532	2	DNA	Wallaby DNA	MSTS01000149	15.37%	837	213
**AC189117**	80.50%	80.50%	217.178	Gallus gallus BAC clone CH261-40E1 from chromosome z, complete sequence	2.60E-51	44.00%	113067	112767	2	DNA	Gallus gallus	MSTS01000015	7.50%	1640	1336
**XM_021000463**	80.50%	80.50%	82.3729	PREDICTED: Phascolarctos cinereus nucleoporin 133 (NUP133), mRNA	9.91E-11	16.60%	4304	4188	2	mRNA	PREDICTED: Phascolarctos cinereus	MSTS01000124	2.68%	1239	1131
**CU468218**	80.30%	80.30%	217.178	Wallaby DNA sequence from clone MEKBa-34H17, complete sequence	2.60E-51	43.80%	150579	150285	2	DNA	Wallaby DNA	MSTS01000045	7.37%	633	335
**CR956372**	79.50%	79.50%	248.571	Wallaby DNA sequence from clone GRWB-15A6, complete sequence	9.17E-61	44.10%	32752	32402	2	DNA	Wallaby DNA	MSTS01000017	8.79%	4040	3686
**CU302372**	79.40%	79.40%	401.843	Wallaby DNA sequence from clone MEKBa-165K22, complete sequence	6.70E-107	46.90%	33898	33316	2	DNA	Wallaby DNA	MSTS01000010	14.35%	2061	1478
**CU012066**	79.40%	79.40%	377.837	Wallaby DNA sequence from clone MEKBa-107A11, complete sequence	1.13E-99	46.50%	44587	44039	2	DNA	Wallaby DNA	MSTS01000120	13.55%	3486	2937
**CU311201**	79.10%	79.10%	156.239	Wallaby DNA sequence from clone MEKBa-426G1, complete sequence	5.76E-33	42.50%	101580	101349	2	DNA	Wallaby DNA	MSTS01000130	5.97%	2657	2415

**CR936538**	78.70%	78.70%	1325.17	Wallaby DNA sequence from clone MEKBa-43B7, complete sequence	0	64.80%	54810	52738	2	DNA	Wallaby DNA	MSTS01000062	50.82%	3956	1889
**AY737497**	78.40%	78.40%	601.281	Macropus eugenii green-sensitive visual pigment (OPN1MW), CXorf2 (CXorf2), and TKT2-like protein (TKT2) genes, complete cds	6.14E-167	51.40%	53953	52972	2	DNA	Macropus eugenii	MSTS01000008	24.46%	2132	1139
**CU468126**	78.20%	78.20%	222.718	Wallaby DNA sequence from clone MEKBa-180L7, complete sequence	5.57E-53	43.50%	22325	21976	2	DNA	Wallaby DNA	MSTS01000022	8.71%	2125	1773
**CP011893**	77.80%	78.00%	704.694	Ovis canadensis canadensis isolate 43U chromosome 8 sequence	0	53.10%	1424643	1423484	2	DNA	Ovis canadensis	MSTS01000032	28.12%	1836	693
**CU463022**	77.40%	77.40%	885.665	Wallaby DNA sequence from clone MEKBa-161M12, complete sequence	0	58.10%	120458	118843	2	DNA	Wallaby DNA	MSTS01000003	38.90%	3416	1836
**FP236650**	77.30%	77.30%	76.8329	Wallaby DNA sequence from clone MEKBa-231N5, complete sequence	4.61E-09	15.30%	22161	22021	2	DNA	Wallaby DNA	MSTS01000215	3.29%	3049	2916
**AL157370**	77.00%	77.00%	193.172	Human DNA sequence from clone RP11-289M23 on chromosome 6, complete sequence	4.39E-44	43.10%	4641	4288	2	DNA	Human DNA	MSTS01000027	9.12%	1923	1553
**FP103010**	76.90%	76.90%	121.153	Zebrafish DNA sequence from clone DKEY-124P11 in linkage group 14, complete sequence	2.10E-22	40.80%	129675	129447	2	DNA	Zebrafish DNA	MSTS01000038	5.70%	1150	919
**CU329700**	76.10%	76.10%	651.141	Wallaby DNA sequence from clone MEKBa-420A22, complete sequence	0	54.90%	45850	44467	2	DNA	Wallaby DNA	MSTS01000151	33.63%	1366	1
**CU468218**	73.20%	73.20%	156.239	Wallaby DNA sequence from clone MEKBa-34H17, complete sequence	5.76E-33	42.50%	108368	107889	2	DNA	Wallaby DNA	MSTS01000303	11.80%	1305	826
